# Using *de novo* transcriptome assembly and analysis to study RNAi in *Phenacoccus solenopsis* Tinsley (Hemiptera: Pseudococcidae)

**DOI:** 10.1038/s41598-019-49997-y

**Published:** 2019-09-23

**Authors:** Satnam Singh, Mridula Gupta, Suneet Pandher, Gurmeet Kaur, Neha Goel, Pankaj Rathore

**Affiliations:** 1 Punjab Agricultural University, Regional Research Station, Faridkot, 151203 Punjab India; 2 Forest Research Institute, Dehradun, Uttaranchal India

**Keywords:** Transcriptomics, Entomology

## Abstract

*Phenacoccus solenopsis* is one of the major polyphagous crop pests in India. Inadequate genomic or transcriptomic resources have limited the molecular studies in this insect despite its huge economic importance. The existing molecular sequence resources of this insect were supplemented through RNA sequencing, *de novo* transcriptome assembly and analysis, which generated 12, 925 CDS from 23,643 contigs with an average size of 1077.5 bp per CDS and 85.1% positive BLAST hits with NCBI Non redundant (nr) database. Twenty three genes involved in RNAi machinery identified through BLASTx search against NCBI nr database suggested the existence of robust RNAi in mealybug. RNAi in *P. solenopsis* was demonstrated through knockdown of *IAP* (Inhibitor of Apoptosis)*, AQP* (Aquaporin)*, CAL* (Calcitonin)*, VATPase* (V-type proton ATPase subunit F 1)*, bursicon, chitin synthase, SNF7* and *α*-*amylase* by injecting sequence specific dsRNA of respective genes in adult female. Additionally, feeding RNAi has been demonstrated in 2^nd^ instar nymph through dsRNA uptake in plant. The knockdown of core RNAi machinery genes such as *Dicer*, *Argonaute* and *Staufen* significantly hampered RNAi efficiency in this insect. However, downregulation of *dsRNases* improved RNAi efficiency. Sequential studies for understanding RNAi in *P. solenopsis* using transcriptome sequences have also been reported. The present study provides a base for future research on developing RNAi as strategy for management of this pest.

## Introduction

*Phenacoccus solenopsis*, commonly known as solenopsis mealybug, is a polyphagous pest, which infests major food and fiber crops in India. It appeared as serious pest of cotton and threatened its cultivation in 2007^1^. Both adults and crawlers suck the sap from different plant parts and cause premature leaf drop and dieback, additionally the honeydew excreted by mealybug serves as a medium for growth of sooty mold which reduces the photosynthetic ability of plants^2^. The major control strategy for this insect relies on the synthetic insecticides^3^, however, their indiscriminate use has given rise to many debatable issues of residues, off-target effects, environmental pollution and development of insecticide resistance by the target pest which necessitate development of ecofriendly pest-management strategies. The successful example is the development of transgenic cotton varieties/hybrids synthesizing Cry proteins and their cultivation for the control of bollworms. However, presently there are no *Bacillus thuringiensis* (Bt) toxins effective against hemipteran insects. Recently some pests have even shown capability of developing resistance against these Bt-based transgenic crops e.g.pink bollworm developing high level of resistance to cotton expressing Cry1Ac and Cry 2Ab proteins from India^4,5^. In this context, focus has to be shifted towards strategies that are less liable to be overcome by insects. RNAi has emerged as a potential tool for studying functional genomics and has wide scope to develop into future insect-pest management strategies based on dsRNA sprays or transgenic plants^6,7^. RNAi has been studied in many insect species^8,9^ and its effectiveness is highly variable among them^10,11^. RNAi involves sequence specific downregulation of the targeted gene through dsRNA feeding or injection in insects, which results in the loss of function of a vital physiological process. The technology can be used for the identification of the novel and vital targets in insects from pest-management perspective and also holds potential in a way that likelihood of insects developing resistance to dsRNA is very meek. The sequence information of genes to be targeted in an organism of interest is the prerequisite for this technology. The molecular studies in *P. solenopsis* are still at infancy due to limited sequence data resources. So the present study was carried out to generate transcriptome sequences data and explore RNAi in this insect.

## Methodology

### De novo transcriptome assembly and analysis

Total RNA was isolated from the mealybug using Direct-Zol RNA miniprep kit (ZYMO Research). RNA-seq paired end sequencing libraries were prepared using TruSeq standard mRNA sample preparation kit (Illumina). Poly A-tailed mRNA fragments were harvested from total RNA using poly-T magnetic beads (Illumina). The purified mRNA was fragmented enzymatically followed by 1^st^ strand cDNA synthesis using SuperScriptII (Illumina) and Act-D mix (Illumina). XP beads (Ampure) were used to purify cDNA followed by A-tailing and adapter ligation, and finally enriched by limited number of PCR cycles. The quality and quantity of PCR enriched libraries were evaluated by 4200 Tape Station system (Agilent Technologies) using High sensitivity D1000 Screen tape (Agilent Technologies). The quantified libraries were subjected to paired end (PE) (2 × 75 bp) sequencing using the NextSeq500 (Illumina)^12^.

High quality clean reads were obtained using Trimmomatic V0.35 after eliminating adapter sequences, ambiguous reads (reads with unknown nucleotides “N” more than 5%) and low-quality sequences (reads with 10% quality threshold (QV) <20PHRED score). These reads were used for *de novo* assembly using default parameters. Adapter trimming, Sliding window, Leading and Trailing were performed, if threshold quality was below 20. Further, the sequences below 75 bp were dropped out after trimming using Minlength^13^. These filtered reads were used for *de novo* assembly of transcripts using Velvet V1.2.10^14^ and Oases V0.2.09^15^ on optimized K mer 57 and 47, respectively. The reads were mapped back to their respective assembled transcripts using Burrows-Wheeler Aligner BWAV0.7.12 for quantitative assessment. Open reading frames (CDS) of all assembled transcripts were predicted using TransDecoder (http://transdecoder.sourceforge.net) and searched against NCBI Non redundant (nr) protein databases using BLASTx.

Gene ontology and functional annotation of predicted CDS was done using Blast2GO^16^. GO terms were retrieved from GO mapping using BLAST x of functionally annotated CDS. CDS were also mapped to reference canonical pathways in the KEGG to determine the possible involvement of predicted CDS in biological pathways. The KEGG Orthology (KO) assignments and corresponding Enzyme Commission (EC) numbers were assigned to annotate and predict CDS using KEGG automated annotation server (KASS) (http://www.genome.jp/kaas-bin/kaas_main). Apart from this, Benchmarking Universal Single-Copy Orthologs (BUSCO v1.1b1) tool was used to measure the completeness and contiguity of the whole transcriptome. This analysis was performed using the early access insect and arthropod BUSCO lineages comprising of 42 insect and 60 arthropod species. The analysis was performed against 1658 insect (http://busco.ezlab.org/v2/datasets/insecta_odb9.tar.gz) and 1066 arthropod (https://busco.ezlab.org/datasets/arthropoda_odb9.tar.gz) BUSCO sets. The available stage specific transcriptome data of *P. solenopsis* (SRR6801044) were also assembled using earlier defined approaches^17^. High quality reads were assembled into contigs using various short read *de novo* assemblers such as Trinity^18^ and CLC Genomics Workbench, which were based on default *k*-mer strategy. To generate super assembly, the contigs from all these transcriptomes were merged and assembled using Overlap-layout-consensus (OLC) based CAP3^19^.

### Exploring RNAi machinery genes

To explore the existence of functional RNAi, the genes involved in RNAi pathway were identified from the functionally annotated transcriptome data of *P. solenopsis* keeping threshold e-value <e^−30^. The super assembly was also used to explore additional or larger fragments of RNAi pathway genes. The FASTQ data files of this submission were filtered and processed as per procedure explained under *de novo* transcriptome assembly and analysis to obtain transcripts. RNAi pathway gene sequences annotated and identified from our transcriptome were searched against the super assembly transcripts using local blast in BioEdit^20^. The predicted RNAi genes of *P. solenopsis* were further validated by searching their homologues in other insect species using BLASTx. Amino acid sequence alignment of *P. solenopsis* core RNAi genes (*Dicer-1*, *Dicer-2*, *Ago-1*, *Ago-2*, *Piwi*, *Aubergine* and *Drosha*) was done using ClustalW with default parameters in MEGAX along with homologs genes from other insect species such as *Zootermopsis nevadensis, Drosophila melanogaster, Amrasca biguttula*, *Tribolium castaneum, Apis mellifera, Bombyx mori, Thrips tabaci* and *Nilaparvata lugens*. Phylogenetic tree was constructed by Maximum Parsimony method using MEGAX at 1000 bootstrap.

### Demonstration of RNAi through dsRNA injection

To validate the predicted RNAi machinery from mealybug transcriptome, eight different genes i.e. *aquaporin* (*AQP*), *calcitonin like receptor* (*CAL*), *inhibitor of apoptosis* (*IAP*), *VATPase* (V-type proton ATPase subunit F 1)*, bursicon, chitin synthase, α-amylase* and *SNF7* (MH712873.1, MK956911-MK956917) associated with different processes were identified from sequenced transcriptome for dsRNA mediated knockdown (Supplementary Information_SI_Table 1). Total RNA was isolated from the whole body tissue of adult mealybug using Trizol (Sigma) as per manufacturer’s instructions. cDNA was synthesized from total RNA (1 µg) using PrimeScript™ 1^st^ strand cDNA Synthesis Kit (Clontech Takara) as per manufacturer’s protocol. The template for dsRNA against each gene was amplified from cDNA using gene specific primers (Primer 3 software^21^) having T7 promoter sequence (TAATACGACTCACTATAGGG) at 5′ end of both reverse and forward primers (Table 1). Amplified fragments of targeted genes were purified using Nucleospin PCR cleanup (Macherey-Nagel Nucleospin Gel and PCR Cleanup) and finally used for dsRNA synthesis with T7 RiboMAX™ Express RNAi System (Promega) following manufacturer’s protocol.

**Table 1:** Primer sequences of template dsRNA for target genes of *Phenacoccus solenopsis*.

Gene	dsRNA Primers	qPCR primers
Calcitonin	5′TAATACGACTCACTATACCTATGGTTGGTATGGTACA3′	5′CGGCGAAGTGATTTCAGCTATT3′
	5′TAATACGACTCACTATAGATCGACGAATGAGGAGTAT3′	5′GTATATGTGGCTGCGTGCTATG3′
Inhibitor of Apoptosis	5′TAATACGACTCACTATAGGTAGAGCATCGTCGTTATTC3′	5′AATAACGTATCCGGCCAAGG3′
	5′TAATACGACTCACTATAGCTTCCTCTCTGATCAAATC3′	5′CGACCGAGTTGGCAGAATTA3′
Aquaporin	5′ TAATACGACTCACTATAGGAATGATCTCGCCGATTAC3′	5′GCTCTACTGGATTGCTCCTTTG3′
	5′ TAATACGACTCACTATAGGATCTTCATCGAGCAAACA3′	5′TAGACGCTTCGCCCATATCT3′
Amylase	5′TAATACGACTCACTATAGCCTTGCCAGATGATTAC3′	5′GGCGATGAACTAGGTATGGAG3′
	5′TAATACGACTCACTATAGGGTGGATCTACTGTTTG3′	5′GGTGTTCTAGCTTTGTCCCT3′
SNF7	5′TAATACGACTCACTATAGGGCCTTCCTTACCGAATAG3′	5′ACCGTAGAAAGCCCGTTTAG3′
	5′TAATACGACTCACTATAGGGGTATGTTATCCGTTGGG3′	5′CATACGCCTGGTGTTTCTATCT3′
Bursicon	5′TAATACGACTCACTATAGGGCCAATACCATCCTTTGC3′	5′CAAGAAAGTGGAGAACGAGAAG3′
	5′ TAATACGACTCACTATAGGGCACATGCATTCTAGAGG3′	5′GCATTCTAGAGGTGCCTTTG3′
dsRNAse	5′TAATACGACTCACTATAGGAAGGCCGATTTCGTCTATG3′	5′GAGACTGGAGTGGCCATAATC3′
	5′TAATACGACTCACTATAGATGGCCACTCCAGTCTCTTT3′	5′TTCGCTGAACTGCGGTAA3′
VATPase	5′TAATACGACTCACTATAGCTCCTTATCCGTTCGGTATC3′	5′GGTCGGCATTCTAGTGTTGA 3′
	5′TAATACGACTCACTATAGCCAAGGAATGCAGATAACAG3′	5′TCCGAGACCACTGTAGAATTTG3′
Chitin Synthase	5′TAATACGACTCACTATAGGGGTGGAGAGTGAAGATAC3′	5′AGGAGAAGGAGGACCAGATAC3′
	5′TAATACGACTCACTATAGGGGTTCGAGTTGGAGATAC3′	5′ACGACGTTCGGTTTGTAGAG3′

Gene functional studies were first validated through preliminary studies taking substantial number of adult mealybugs (25–30) for each gene. Thereafter the final studies were conducted with adult mealybugs in three biological replicates (5 insects/replicate) injected individually with 10 µg of gene specific dsRNA from dorsal side using glass capillary (#3-000-203-G) (Drummond Scientific, Broomall, PA) and Nanojet^TM^ (Drummond Scientific)^22^. The same quantity of dsGFP was injected in control insects. Immediately after injection the insects were placed on ice for five minutes to avoid injection stress and finally released on cotton leaves in environmental chamber at 70% RH and 25 ± 2 °C temperature. Live insects were collected in Tri-Reagent^®^ (Sigma-Aldrich) for RNA extraction after 72 h as per manufacturer’s instructions. mRNA levels of knocked down genes were quantified in Lightcycler® (Roche, USA) using respective cDNA (First strand cDNA synthesis kit, ThermoFisher Scientific) and Syber ExcelTaq™ 2X Q-PCR Master Mix (SMOBIO) as per manufacturer’s protocol. Each qPCR reaction constituted of 1:10 diluted 1 µl of cDNA, 0.2 µl of gene-specific primers (10 mM) (Table 1) and 5 µl of SYBR premix and rest nuclease free water to make final volume of 10 µl. The relative expression level of each gene was estimated using ∆∆CT method after normalization with *28* *s* (MH712871.1) as reference gene^22^.

### Physiological validation of *AQP* and *CAL* knockdown through fluid loss assays

The effect of dsAQP and dsCAL was also evaluated through post injection fluid loss estimation as these genes play a key role in osmoregulation in insects^23,24^. Adult mealybugs (n = 3) in three replicates were injected with 10 µg dsRNA against *AQP* and *CAL* and released on water sensitive paper (Teejet Technologies, USA) disc (5 cm diameter) for 12 h. The fluid excretion from insect body yields blue colour dots on water sensitive paper, which was used for qualitative estimation of fluid loss from the insect. For quantitative estimation of water excretion, four individual replicates in each treatment were injected with 10 µg of dsAQP and dsGFP and released into Water Loss Monitoring System consisting of RM-8 Flow Multiplexer, RH-300 Relative Humidity analyzer, SS-4 gas analyzer sub-sampler pump and mass flow meter, RC-chambers and UI-3 A/D interface and ExpeData software (Sable Systems International, USA). Before releasing insects into this chamber, dry N_2_ air was passed through chambers for 1 hour. Data on fluid excretion was acquired by ExpeData software (Sable systems). The data was recorded for 1 h in each treatment and water excretion was calculated using formula VH2O = FR*dh2o/(1-Fewv), where Fewv = WVP/101.3.

### Evaluation of impact of *chitin synthase* and *bursicon* knockdown on mealybug

*Chitin synthase* is indispensable for chitin synthesis, while *bursicon* has role in melanization and sclerotization of cuticle. Both these genes are related with molting process and the phenotypic changes associated with their knockdown can be well observed in early stages compared to adults. Thus five 3^rd^ instar insects each were injected with 10 µg dsRNA against *chitin synthase* (dsChsyn) and *bursicon* (dsBur) separately and released on cotton leaves as per earlier explained methodology. The insect were observed 72 h after injection and compared with dsGFP injected insects for phenotypic changes. The size of each insect was measured under the Stereo Zoom Microscope (Olympus SZX7).

### Calculation of LD_50_ using Probit analysis

Mortality studies in mealybug after injection of dsRNA against *SNF7*, *IAP, VATpase* and *α-amylase* were performed at multiple doses to calculate the dose-mortality relationship using Probit analysis^25^ in Polo software^26^. Different doses, i.e., 10, 20, 40, 60 and 80 µg of dsSNF7, dsIAP, dsVATpase, dsAmylase and dsGFP were injected into adult mealybug (n = 5) with three replications. The insects were released on cotton leaves after injection to record the mortality after 48 h.

### Demonstration of feeding RNAi through petiole dip assay

**Figure 1:**
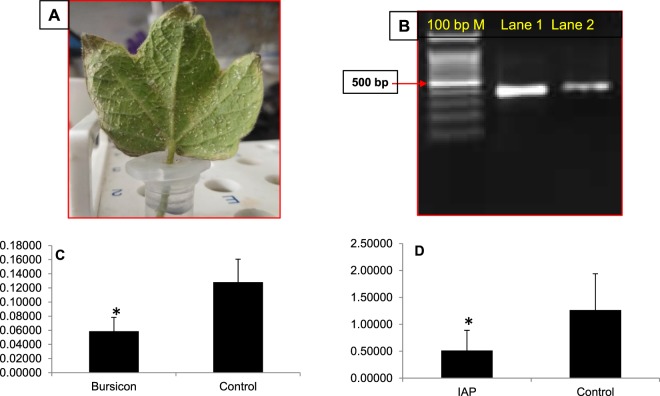
Feeding RNAi in mealybug with 380 µg of dsRNA imbibed in leaf using petiole dip assay. (**A**) Experimental setup for mealybug feeding. (**B**) Uptake and stability of dsRNA extracted from leaf visualized on agarose gel (1%) Lane1: Crude dsGFP (+ve control) Lane 2: dsRNA isolated from the leaf after uptake. Relative expression of *bursicon*and *IAP *in *Phenacoccus solenopsis* 48 h post feeding in comparison to GFP control: (**C**) *Bursicon* (54.1% knockdown) (**D**) *IAP* (59.4% knockdown). The expression level of each gene has been normalized with 28 s as internal control. The error bars represent the standard deviation (3 replicates) and *represents significant differences in mRNA transcripts compared to GFP control (P ≤ 0.05, Student’s t-test).

To study the feeding RNAi in the *P. solenopis*, 380 µg dsRNA of *Bursicon*, *IAP* and GFP was made to imbibe in leaves through petiole dip assay (Fig. 1(A)) followed by release of second instar mealybug nymphs on it and dipping the leaf petiole in nuclease free water for 48 h. Mealybug nymphs were collected (n = 25 in 3 biological replicates) from leaves 72 h post release for isolation of total RNA using Tri-Reagent^®^ (Sigma-Aldrich)) followed by cDNA synthesis (First strand cDNA synthesis kit, ThermoFisher Scientific) and relative estimation of mRNA transcripts of targeted genes using RT-qPCR as described earlier. dsRNA was extracted from leaf post petiole dip (12 h) to confirm its uptake in leaf as per earlier described methodology^27^. Additionally, membrane feeding with 500 ng dsRNA per µl of artificial diet (Sucrose 30% solution, table sugar 50 mg and 1 ml yellow food dye (Ajanta Food Products Co., Solan, India) stretched between two layers of parafilm was tried in 2^nd^ and 3^rd^ instar, and female adult mealybug (Supplementary Information 4:Fig. 1). Except 2^nd^ instar membrane feeding could not be achieved in any other instar or insect stage. This may be attributed to low adaptability of third instar and adult female mealybug while shifting from natural plant host to artificial diet compared to 2^nd^ instar.

### Functional validation of core RNAi machinery genes through their knockdown

Core RNAi machinery genes such as *Ago-2* and *Dicer-2* were knocked down to evaluate their effect on RNAi efficiency of dsAQP injected adult mealybug. dsRNA was synthesized against *Ago-2*, *Dicer-2* and *AQP* based on sequences retrieved from the transcriptome data using standard procedures as explained in earlier section. dsRNA (10 µg) against two RNAi pathway genes along with 10 µg of dsGFP was injected separately in five adult mealybug individuals in triplicates followed by injection of 10 µg of dsAQP after 24 hours of first injection as per procedure mentioned in Supplementary Information 4_SI_Fig. 2. Immediately after injection, the insects were placed on ice for five minutes. Treated insects were released on cotton leaves and after 48 h of second injection, total RNA was isolated from live insects followed by cDNA synthesis as per procedure explained earlier. mRNA level of *AQP* was quantified from different treatments in Lightcycler® (Roche, USA) using respective cDNA and SYBR Excel Taq™ 2X Q-PCR Master Mix (SMOBIO) as explained earlier.

Similarly, the role of *Staufen* in RNAi was also studied in mealybug. Previous reports state that *Staufen C*, dsRNA binding domain has a major role in RNA interference in coleopterans^28^. Thus, 10 µg dsRNA against mealybug specific *Staufen* was injected along with dsGFP (10 µg) separately in biological triplicates (n = 5) followed by injection of 10 µg of dsSNF7 and dsIAP after 24 h separately to each set of earlier injected insects as per procedure explained in Supplementary information 4_SI_Fig. 2. The insects were released on cotton leaves and after 48 h of second injection, total RNA was isolated from live insect followed by cDNA synthesis as per procedure explained earlier. mRNA level of *SNF7* and *IAP* were quantified from different treatments in Lightcycler® (Roche, USA) using respective cDNA and SYBR Excel Taq™ 2X Q-PCR Master Mix (SMOBIO) as explained earlier.

### Degradation of dsRNA in mealybug body fluids and knockdown effect of *dsRNases* on RNAi efficiency

Adult mealybugs (n = 20) were collected and held on wax tray with entomological pins. With the help of fine blade, cut was made precisely to collect the body fluids (hemolymph + other body liquids) from the insect. To avoid coagulation of proteins 1 mg of phenylthiourea (Hi-media) was added to the crude fluid in 1.5 ml tube. The fluid was centrifuged (10,000 rpm for 5 minutes), aspired and serially diluted into 1/10, 1/100, 1/1000 using nuclease free water. dsRNA (dsGFP) (1 µg) was incubated in each dilution of crude fluid at room temperature for 1, 3 and 5 h and observed on agarose gel at each time interval. Additionally, the effect of *dsRNases* gene knockdown on RNAi efficiency was studied by injecting *dsRNases* specific dsRNA (dsdsRNase) followed by injection of dsRNA against the target genes i.e. *IAP* and *SNF7* as per methodology explained in the Supplementary information 4_SI_Fig. 2. For this 10 µg of dsRNA against *dsRNases* and equal amount of dsGFP was injected separately in adult mealybug individuals (n = 5) in triplicates. After 24 h, 10 µg dsIAP and dsSNF7 each was separately injected to earlier treated bugs. Post-injection, the insects were released on the cotton leaves followed by RNA isolation after 72 h using Tri-Reagent^®^ (Sigma-Aldrich) and cDNA synthesis (First strand cDNA synthesis kit, ThermoFisher Scientific) for relative expression of mRNA transcripts using RT-qPCR as explained earlier.

## Results and Discussion

*P. solenopsis* is an important cotton pest. Although full-genome information is lacking for *P. solenopsis*, our transcriptome assembly may represent a significant proportion of the functional genes in this specie. The deep coverage of *de novo* transcriptome of the insect was obtained from cDNA libraries using Illumina paired-end (2 × 75 bp) sequencing technology. Contaminated reads with low quality and primer/adaptors were removed from these transcriptomes to obtain quality reads. A total of 19,366,549 high-quality reads were assembled into 23,643 transcripts with average length of 875.56 nt per contig. Assembly of the available transcriptomes of *P. solenopsis* discovered the presence of 55198 transcripts in second instar (SRR6782025), 55569 transcripts in adult (SRR6782023), 49193 transcripts in third instar (SRR6782022) and 64497 in the eggs (SRR6782024). Additionally, the super assembly of all these transcriptomes generated 27,991 common contigs, which validated the RNAi pathway and RNAi targets identified from the present *P. solenopsis* transcriptome (SRR6801044).

**Figure 2:**
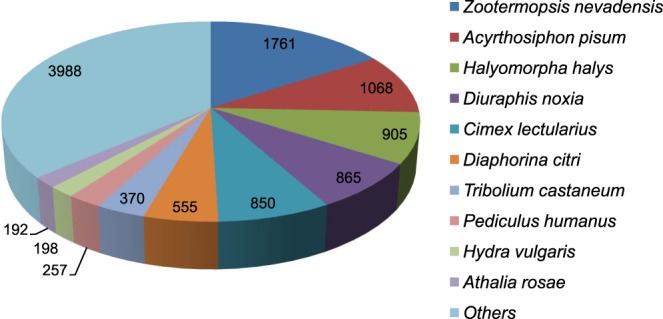
Top BLAST hits of *Phenacoccus solenopsis* coding sequences against other insect species based on highest score in BLASTx using homology searches against nr database.

A total of 12,925 CDS were predicted using TransDecoder with size ranging between 297 and 13,776 bp with average length of 1077.5 bp (Table 2). Predicted CDS were annotated against NCBI nr database using BLASTx tool. From a total of 12,925 CDS, 85.1 per cent (11,009) showed positive blast hits matches with insects such as *Z. nevadensis, Acyrthosiphon pisum*, *Halyomorpha halys*, *Diuraphis noxia*, *Cimex lectularius*, *Diaphorina citri*, *Tribolium castaneum*, *Pediculus humanus*, *Hydra vulgaris* and *Athalia rosae* (Fig. 2).

**Table 2:** Transcriptome statistics of sequenced total RNA of *Phenacoccus solenopsis*.

	Statistics
No. of Reads	19,366,549
Number of bases	2,918,088,371
Total data in Gb	~3
No. of Transcripts	23,643
Total transcript length(bases)	20,700,870 bp
N50	1,334 bp
Maximum transcript length	13,786 bp
Minimum transcript length	200 bp
Mean transcript length	875.56 bp
No. of CDS	12,925
Total CDS length(bases)	13,926,924 bp
Maximum CDS length	13,776 bp
Minimum CDS length	297 bp
Mean CDS length	1077.518 bp
**Range of transcript (bases)**	
200 ≤ transcript < 500	10,582
500 ≤ transcript < 1000	6,256
1000 ≤ transcript < 2000	4,668
2000 ≤ transcript < 3000	1,470
3000 ≤ transcript < 4000	416
4000 ≤ transcript < 5000	151
transcript ≥ 5000	100
**Range of CDS (bases)**	
200 ≤ CDS < 500	3,235
500 ≤ CDS < 1000	4,601
1000 ≤ CDS < 2000	3,642
2000 ≤ CDS < 3000	961
3000 ≤ CDS < 4000	306
4000 ≤ CDS < 5000	102
CDS ≥ 5000	78

The predicted CDS from *P. solenopsis* transcriptome showed the highest BLAST hits with *Z. nevadensis*. This may be an indicative of biological genomic conservation as both the species are classified under Infraclass- Neoptera. However, the number of BLAST hits with ‘Query sequences’ will also depend on the availability of related ‘Subject sequences’ in the databases (NCBI). Present study has been successful in retrieving comparable number of predicted CDS i.e. 12,925 as reported in earlier sequenced transcriptomes from other insect species such as 14,797 CDS in *Drosophila melanogaster*^29^, 11,056 in *Cloeonviridulum*^30^, 18,501 in *B.mori*^31^ and 18,071 in *Plutella xylostella*^32^.

**Figure 3:**
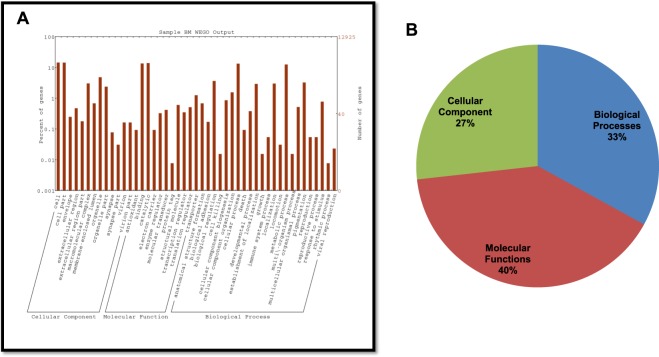
(**A**) Classification of *Phenacoccus solenopsis* CDS associated with cellular component, molecular function and biological process based on predicted Gene Ontology terms via WEGO plot. GO terms were determined using Blast2Go with an e-value cutoff of 10^−5^. (**B**) CDS categorized by Gene Ontology using Blast2Go under different processes of *Phenacoccus solenopsis*

Gene ontology of *P. solenopsis* transcriptome exposed 7156 CDS GO categories under Biological Processes (2361), Molecular Function (2880) and Cellular Component (1915) (Fig. 3). The most enriched terms of Biological processes were organic substance metabolic process (11.6%), primary metabolic process (10.99%) and cellular metabolic process (10.93%) (Fig. 3). In Molecular function, the major contributions were towards the organic cyclic compound binding and heterocyclic compound binding (10.6%). Under Cellular component, 9.6% sequences comprised of intracellular and 8.03% in intracellular part.

**Figure 4:**
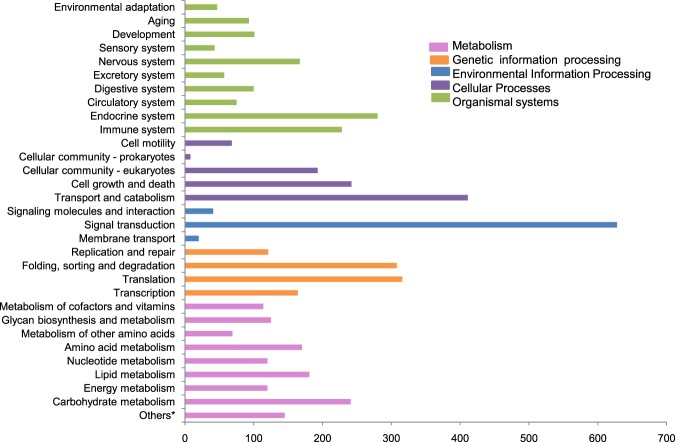
KEGG pathway classification of CDS generated from *Phenacoccus solenopsis* transcriptome. *Others represents overall count for the following pathways: Carbon metabolism, 2-Oxocarboxylic acid metabolism Fatty acid metabolism, Biosynthesis of amino acids, Degradation of aromatic compounds.

KEGG analysis for functional annotation was done to assign the transcripts KO IDs under four biological processes, i.e., Metabolism, Cellular, Genetic information processing and Environmental information (Fig. 4). Only 4,996 CDS (38.6%) out of 12,925 were annotated, assigned the KO IDs for KEGG pathways and were categorized in 31 different functional KAAS pathway categories. The majority of CDS grouped into signal transduction (12.5%) followed by transport and metabolism (8.2%), translation (6.3%) and carbohydrate metabolism (4.8%).

The completeness and quality of *P. solenopsis* transcriptome evaluated by BUSCO analysis indicated that 79.1 and 86.2% BUSCO genes were “complete” compared to insect and arthropod lineages, respectively (Table 3). Orthologs are said to be ‘Complete’ if the length of their aligned sequence fall within two standard deviations (2s) of the BUSCO group’s mean length (i.e. 95% expectation), otherwise they are grouped into ‘Fragmented’ recoveries. Some transcriptome assemblies appearing less complete than their corresponding gene sets reveal the limitations of BUSCO gene prediction step. Alternatively, a reversal of this trend suggests that the annotated gene set may be missing some BUSCO gene matches that are in fact present in the transcriptome. The ‘complete’ genes found with more than single copy are designated as ‘duplicated’ and these should be rare because recovery of too many duplicates is indicative of erroneous assembly as BUSCOs evolve under single-copy control^33^. Both ‘fragmented’ and ‘missing’ BUSCOs, were low in this transcriptome. Out of the complete BUSCOs only 1.7 and 1.2% were duplicated compared to insect and arthropod lineages, respectively. Thus in BUSCO analysis recovery of ‘Complete’ and single copy complete BUSCOs is indicative of good transcriptome assembly and our results were comparable to the earlier assemblies listed by Simao *et al*.^34^.

**Table 3:** BUSCO statistics for *Phenacoccus solenopsis* transcriptome assembly against insect and arthropod lineages.

BUSCO Notation	Insect	Arthropod
Complete BUSCOs (C)	79.1%	86.2%
•Complete and single-copy BUSCOs (S)	77.4%	85.0%
•Complete and duplicated BUSCOs (D)	1.7%	1.2%
Fragmented BUSCOs (F)	13.6%	9.8%
Missing BUSCOs (M)	7.3%	4.0%
Total BUSCO groups searched (n)	1658	1066

### Existence of RNAi machinery in mealybug

**Figure 5:**
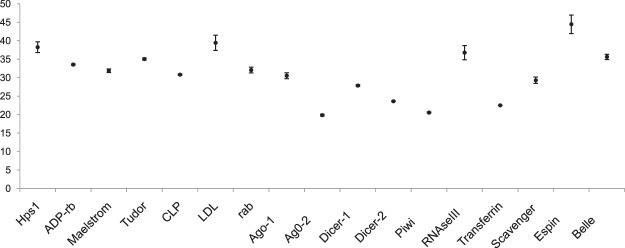
Expression level of RNAi genes in adult *Phenococcus solenopsis* based on Ct values generated through RT-qPCR. Each data point represents the Mean ± S.D. of Ct values based on biological triplicates.

**Figure 6:**
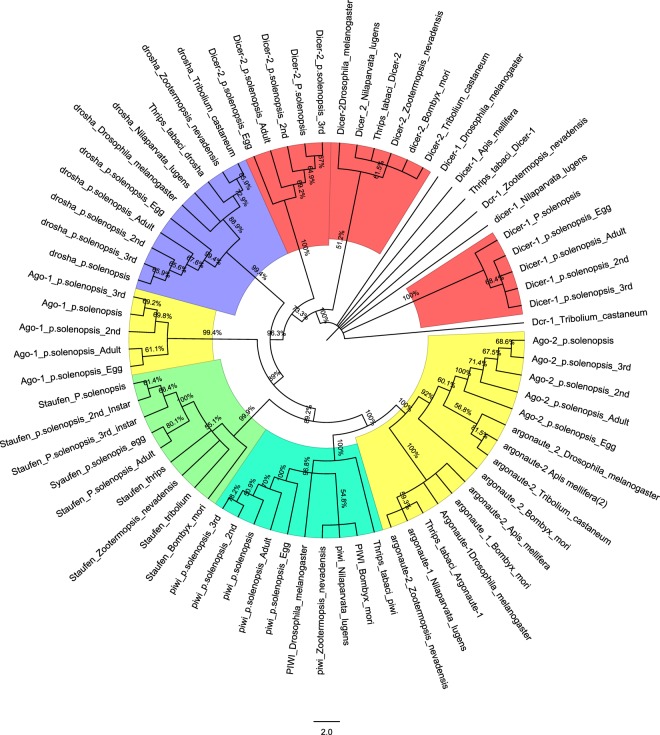
Phylogenetic analysis to predict genetic relatedness of core RNAi genes of *Phenacoccus solenopisis* with other insect species using Maximum Parsimony Method in MEGAX (Bootstrap 1000).

Total of 23 RNAi genes were identified in functionally annotated transcriptome. We explored basic set of RNAi genes from the *P. solenopsis* transcriptome using homologs of *Z. nevadensis*, *H. halys*, *Locusta migratoria* and *C. lectularius* (Supplementary information 4_SI_Table 2). The major genes identified were *Argonaute-1* (*Ago-1*), *Argonaute-2* (*Ago-2*), *Dicer-1*, *Dicer-2*, *RNAse III*, *Piwi* and *Clathrin*. RT-qPCR studies further confirmed the presence of these genes and their variable expression in adult mealybug (Fig. 5). A phylogenetic tree of core RNAi gene sequences identified in mealybug with their homologs from other organisms validated their origin and relationship with RNAi pathway (Fig. 6). RISC factors such as *Epsin*, *Transferrin like receptor1*, *Scavenger receptor1*, *Low density lipoprotein receptor*, *VATPase 16 kDa* (*Vh16a*), *rab-7a*, *ADP-ribosylation factor 6*, *abnormal spindle*, *RNA binding protein fox-*1, *small RNA degrading nuclease 5*, *Hermansky-Pudlak syndrome 3*, *DEAD-box helicase Dbp80*, *arginine N-methyltransferase 1*, *Tudor*, *Clp protease* and RISC loading complex were also identified from this transcriptome (Supplementary information 4_SI_Table 2). However, we could not locate homolog sequences for *SID-1* (*systemic RNA interference defective-1*) and *SID-2* (*systemic RNA interference defective-2*), which are responsible for dsRNA uptake in many species^35,36^. Absence of these two genes in *D. melanogaster* is considered as possible reason for poor RNAi in this insect^8,37,38^. However, the function of *SID* genes in RNAi mechanism is still not clearly known^35^ and some studies relate the dsRNA uptake with the help of *rab-7a*, *Vh16a*, *Scavenger receptor* and *Clathrin* through endocytosis in *D. melanogaster*^39^. Similar genes were also identified from *P. solenopsis*, which may be responsible for dsRNA uptake in this insect. We identified two *Dicer* paralogues i.e. *Dicer-1* and *Dicer-2*, and *RNAseIII*, which mediate dsRNA processing for the formation of siRNA. Like other insect species, we were unable to find RNA-dependent RNA polymerase, even at less significant e-value (e^−3^)^40^ in this insect.

### RNAi of candidate genes and their knockdown effects on mealybug

**Figure 7:**
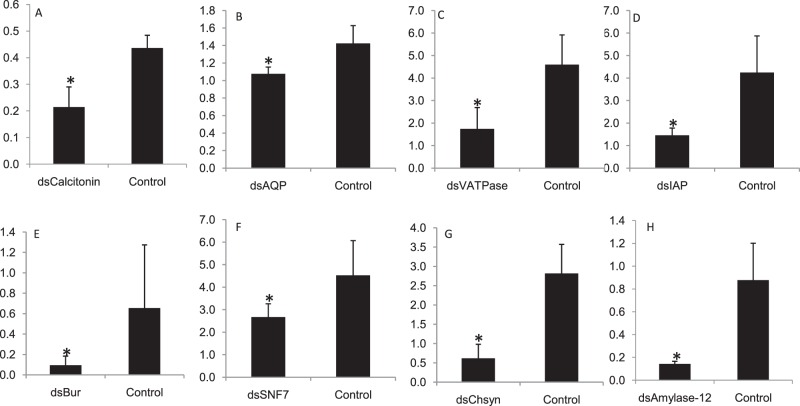
Relative expression of candidate genes in *Phenacoccus solenopsis* 72 h post injection of respective 10 µg of dsRNA in comparison to GFP control (**A**) *calcitonin *50.9% kd (knockdown), (**B**) *aquaporin *(24.4% kd), (**C**) *VATpase* (61.99% kd), (**D**) *IAP* (Inhibitor of Apoptosis) (65.58% kd) (**E**) *bursicon*(85.7% kd), (**F**) *SNF7* (40.8% kd), (**G**) *chitin synthase* (78.02% kd), (**H**) *α-amylase* (83.6% kd). The expression level of each gene has been normalized with 28 s as internal control. The error bars represent the standard deviation (3 replicates) and *represents significant differences in mRNA transcripts compared to GFP control (P ≤ 0.05, Student’s t-test).

**Figure 8:**
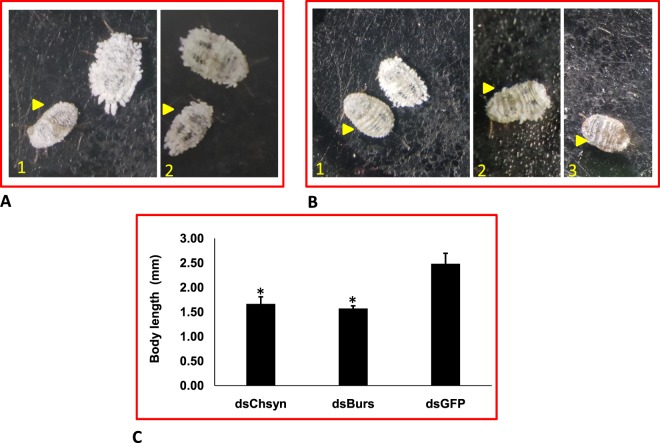
Morphological changes associated with injection of 10 µg dsBurs and dsChsyn in 3^rd^ instar mealy bug compared to dsGFP post 72 hours injection. (**A**) dsBurs injected nymph (yellow pointer) showing reduction in size and low wax coating compared to GFP control (**B**-1) dsChsyn injected nymph compared to GFP control post 72 h (**B**-2, 3) Deformed nymph and reduction in size along with browning post 96 h (**C**) Reduction in length (mm) in dsChsyn and dsBurs treated insects post 72 h compared to dsGFP control insect. The error bars represent standard deviation (n = 5) and *represents significance (P ≤ 0.05, Student’s t-test).

**Figure 9:**
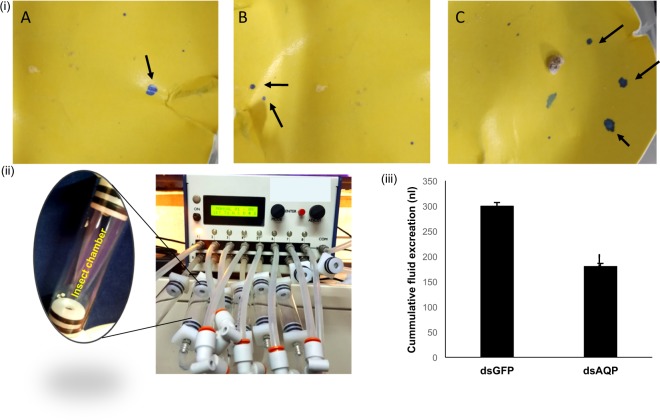
(i) Quantitative and qualitative estimation of fluid loss from mealybug post dsRNA injection (10 µg) against *AQP* and *CAL* as compared to GFP. Qualitative estimation of fluid loss based on water sensitive paper indicating less blue dots (=less fluid loss) in *AQP* and *CAL* as compared to GFP control: (**A**) dsAQP injected (**B**) dsCAL injected (**C**) dsGFP injected (ii) Water vapor analyzer used for evaluation of fluid loss by insect. (iii) Quantitative fluid excretion from mealybug post dsAQP injection compared to dsGFP. The error bars represent standard deviation (n = 4) and *represents significance (P ≤ 0.05, Student’s t-test).

**Figure 10:**
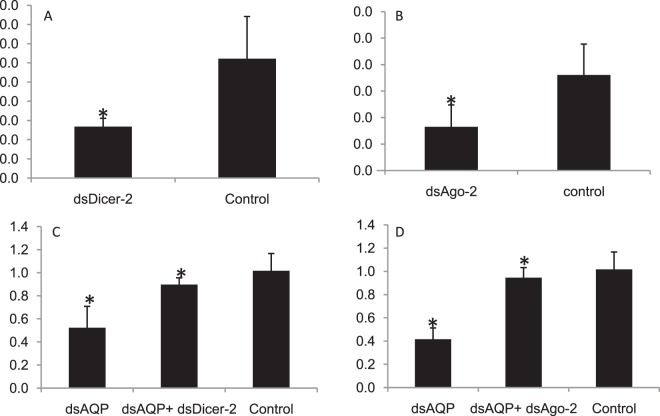
Influence of consecutive knockdown of core RNAi genes on RNAi of *AQP* in *Phenacoccus solenopsis* through dsRNA (10 µg) injection: Relative expression of (**A**) dsDicer-2 (56.9% kd (knockdown)) (**B**) dsAgo-2 (54.22% kd). (**C**) dsAQP + dsDicer-2- *AQP* expression was hampered by 75.6% (**D**) dsAQP + dsAgo-2 (*AQP* expression was hampered 88.01%). The expression level of each gene has been normalized with 28 s as internal control. The error bars represent the standard deviation (3 replicates) and *represents significant differences in mRNA transcripts compared to GFP control (P ≤ 0.05, Student’s t-test).

Gene silencing was performed to substantiate the concept of using transcriptome analysis for discovery of novel RNAi targets. We explored 48 RNAi targets in this transcriptome data, which have been identified or studied in other insect species^41,42^ (Supplementary information 4_SI_Table 3). The expression of target genes (*CAL*, *AQP*, *VATPase*, *IAP, bursicon, chitin synthase, α-amylase* and *SNF7*) was evaluated in all developmental stages and their knockdown was achieved by injecting their respective dsRNA (Supplementary information 4 _SI_Fig. 3). Injection of 10 µg of dsRNA against these genes showed significant knockdown in mRNA levels of target gene compared to dsGFP control insects 72 h post injection. Reduction in mRNA levels was 2.03, 1.32, 2.63, 2.90, 6.99, 1.68, 4.55 and 6.13 fold in *CAL*, *AQP*, *VATPase*, *IAP, bursicon, SNF7, chitin synthase* and *α-amylase*, respectively compared to control insects (Fig. 7). The reduction in mRNA transcripts of particular gene within an insect is regulated by many factors such as dsRNA quantity, insect stage, length of dsRNA, type of gene, dsRNA delivery mode etc^43,44^. In present studies the quantity of dsRNA, insect stage and length of dsRNA (~400 bp) was uniform, which implies that RNAi may vary from gene to gene in the same insect species. Feeding dsRNA through petiole dip assay caused 2.18 and 2.46 fold reduction in mRNA levels of *bursicon* and *IAP* compared to GFP control (Fig. 8). However, feeding of dsBurs (500 ng/µl diet) through membrane caused only 1.4 fold reduction in mRNA levels compared to GFP control. Petiole dip assay was more practical approaching for feeding RNAi in mealybug, thus this method was preferred over membrane feeding (Supplementary Information 4 Fig. 2). The variation in RNAi/efficacy of dsRNA either through feeding or injection may be attributed to the nucleases, which cause the degradation of dsRNA in the hemipteran guts^43^. Our studies report high variation in the knockdown efficiency of *bursicon* between injection and feeding. This could be possibly due to dsRNA dose variation, which could not be ascertained in insect fed on leaves in petiole dip assay. The genes in current studies play vital role in various physiological processes of insect, thus interruption of these processes may be helpful for identification of potential target(s) for the future management of this insect. *CAL* and *AQP* are the osmoregulatory genes required for maintaining water homeostasis. *AQP* is trans-membrane protein which helps in water loss and excretion from the insect^45,46^. Aquaporins are localized in the filter chamber of hemipteran insects (e.g. *Bemisia tabaci*) and found to be associated with rapid water excretion and osmoregulation^47^. *AQPs* also play known roles in heat tolerance in female tsetse flies *Glossina morsitans morsitans* as they maintain water homeostasis^24^. Knockdown effect of osmoregulatory genes visualized with the help of water sensitive paper and water vapor analyzer revealed low fluid excretion in mealybugs injected with dsAQP and dsCAL compared to dsGFP control insects. The qualitative assessment showed that the number and size of the blue dots on water sensitive paper were more/large in dsGFP compared to dsAQP and dsCAL (Fig. 9(i)). The quantitative assessment of cumulative mean fluid loss from the individual mealybug injected with dsAQP was 180 nl in one hour compared to 300 nl in dsGFP control insects (Fig. 10(iii)). Earlier studies with knockdown of *AQP* in *B. tabaci* resulted in significant reduction in fluid excretion compared to dsGFP control as assessed by water sensitive paper assay^48^. Since calcitonins are involved in diuresis in insects^49^, the knockdown of *Aedes aegypti* calcitonin receptor *AaegGPRCAL1* causes significant reduction in fluid excretion as compared to dsGFP control insects estimated using insect humidity chamber^50^ and improved mosquito desiccation resistance^23^. *VATpase* is the proton pump responsible for energizing the plasma membrane and helps in transport of Na+/K(+)-ATPase in epithelial membrane^51^. *VATpase* is one of the frequently used gene for RNAi studies in insects as well as insect cell lines^35,52–54^. Additionally, earlier studies on mosquito larvae revealed that RNAi-based *VATPase* suppression led to adverse effects on health of the insect as it showed elevated Cry11a toxin hypersensitivity^55^. The present studies also achieved 61.9 per cent knockdown in *VATpase* from mealybug using 10 µg of dsRNA^35^. However no phenotypic/morphological abnormality was observed at 72 and 96 h after dsVATpaseinjection compared to control. In mealybug, the maximum knockdown was achieved in *bursicon, chitin synthase* and *α-amalyase* was 85.71, 78.02 and 83.69 per cent, respectively. Knockdown of *bursicon* caused varied level of reduction in insect size and wax deposition in all the tested insects compared to GFP control 72 h post injection (Fig. 8A). The mean reduction in insect length was 36.7% in dsBurs injected insects compared to control (Fig. 8C). Similarly, knockdown of *chitin synthase* also led to reduction in insect size (32.9%) and reduced wax coating in all the tested insects compared to GFP control 72 h post injection. Besides these, mild deformities and browning of insect were also observed at 96 h post injection in some insects compared to GFP control insects (Fig. 8 B-2 and B-3). *Bursicon*, known as insect tanning hormone, has vital role in development of insect cuticle^56,57^. Earlier studies have suggested that RNAi mediated knockdown of *AmBurs α* and *AmBurs βbursicon* genes in *Apis mellifera* prevented the complete formation and tanning of the adult cuticle^56^. Its role in wing expansion was also validated through RNAi-mediated silencing of *bursicon* in silkworm^58^. *chitin synthase* has a prominent role in chitin formation, which is responsible for insect molting and development^59,60^. RNAi mediated knockdown of *chitin synthase* has resulted in insect abnormalities and varying level of mortality in *Phthorimaea operculella*^61^, *Leptinotarsa decemlineata*^62^ and *Anthonomus grandis*^63^. The downregulation of *α-amylase* gene leads to retardation in the growth and development of insect^64^. α-amylase is a digestive enzyme, having role in absorption of nutrients in insects. However, in case of mealybug no morphological changes were observed with the knockdown of *α-amylase*. Knockdown of two *amylase* genes in Pacific oyster *Crassostrea gigas* also resulted in reproductive knockdown phenotypes for both sexes, reduction in gonad area and germ cell under-proliferation^65^. In-depth studies are still required in order to validate dsRNA dose for achieving the desired phenotype in *VATpase*andα-*amylase*. *IAP*^60,66,67^ and *SNF7*^54^ knockdown through RNAi has been evaluated in many insect species. *IAP* proteins have potential role in restriction of the apoptosis in the insect^68^, while *SNF7* functions as a part of the ESCRT (Endosomal Sorting Complex Required for Transport) pathway which plays a crucial role in cellular housekeeping by internalization, transport, sorting and lysosomal degradation of transmembrane proteins. Numerous studies provide evidence which indicate that injection or feeding of dsRNA against *IAP* led to notable mortality in the host insect^52,66^. *DvSnf7* from *Diabrotica virgifera* has shown to be an effective target as *DvSnf7* RNAi leads to lethality of its larvae^44^. The lethality of dsIAP, dsSNF7, dsAmylase and dsVATpase injection in *P. solenopsis* has been studied through dose-mortality relationship achieved through Probit analysis (Supplementary information 4_SI_Fig. 4). It was observed that 10 µg of dsRNA failed to cause any mortality in mealybugs, however reduction in mRNA transcripts of respective gene was observed in all studied genes. The LD_50_ for dsSNF7 was 49.74 µg/insect compared to 43.83 µg/insect for dsIAP, which clearly indicates that higher dose of dsRNA against *SNF7* is required to cause 50% mortality of test insects. Similarly the LD_90_ for these two genes ranged between 81.95 and 85.27 µg/insect (Supplementary information 4_SI_Table 4). The dose mortality relationship couldn’t be calculated for dsAmylase and dsVATpase because of negligible mortality at 20 to 80 µg dsRNA.

The dsRNA mediated knockdown of different genes suggests that impairment in these genes or other vital targets through RNAi may be helpful in identification of potential targets for developing new generation insect management tools.

### RNAi of core RNAi pathway genes

**Figure 11:**
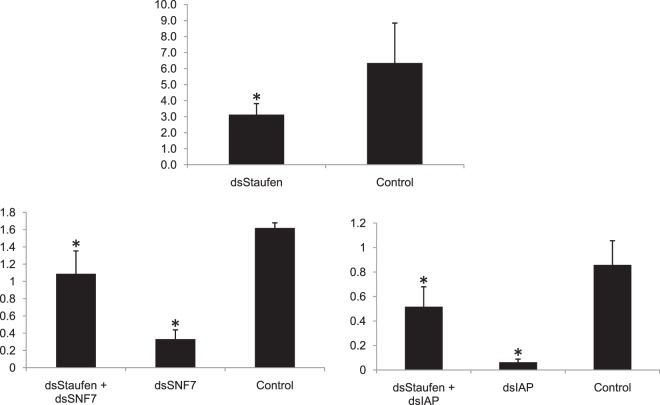
Influence of consecutive knockdown of *Staufen* on RNAi of *AQP* and *IAP* in *Phenacoccus solenopsis* through dsRNA (10 µg) injection (**A**) dsStaufen (50.7% knockdown) (**B**) *SNF7* expression in dsStaufen + dsSNF7 compared to dsGFP and dsSNF alone. (**C**) *IAP* expression levels in insects injected with dsStaufen + dsIAP compared to dsGFP and dsIAP alone. The expression level of each gene has been normalized with *GSTD2* as internal control. The error bars represent the standard deviation (3 replicates) and *represents significant differences in mRNA transcripts compared to GFP control (P ≤ 0.05, Student’s t-test)

We verified the role of *Ago-2, Dicer-2* and *Staufen* in RNAi by consecutive knockdown of these genes followed by *AQP* in adult mealybug insects. The knockdown of *AQP* was significantly reduced in the insects injected with dsAgo-2 and dsDicer-2 as compared to GFP. The RNAi efficiency was reduced to 88.01 and 75.6% in case of dsAQP + dsAgo-2 and dsAQP + dsDicer-2 injected mealybugs, respectively (Fig. 10). Present studies suggest that knockdown of *Dicer-2* and *Ago-2* interfered with RNAi of *AQP* in mealybug. Three distinct *dicer-2* transcripts were retrieved from *P. solenopsis* transcriptome, however characteristic core Dicer domains were common to all. Similarly, eight distinct *Argonaute-2* sequences were also found in this transcriptome. Knockdown of both genes (*Dicer-2* and *Ago-2*) partially blocked RNAi, which was indicative from the mRNA expression level of *AQP* after dsDicer-2 and dsAgo-2 injection. *Dicer-2* is an important component of RISC activation complex and its role has been well studied in siRNA/miRNA silencing pathway of *Drosophila*^69^. *Argonautes* are also known to play a vital role in RNAi in *Drosophila*^70^. Studies have demonstrated the role of *Argonaute* and *Dicer-2* in RNAi, as silencing of these genes led to interruption of RNAi in coleopteran cell line (Lepd-SL1)^35^. In addition, *Dicer-1* has also shown a notable role in siRNA/miRNA machinery pathway in this insect^69^. Besides these genes *Staufen*, a dsRNA-binding protein initially reported from *Drosophila*^71^ has also been identified from *P. solenopsis*. About 56.9–58.7% of RNAi efficiency was reduced when dsStaufen was injected in combination with dsIAP and dsSNF7 (Fig. 11). In *C. elegans*, *Staufen* has been known to play a role in RNAi and recently, robust RNAi efficiency in beetles has been attributed to coleopteran-specific *Staufen* (*StauC*), which is absent in other insect orders^28,72^. Recent study with *Thrips tabaci* also suggest the role of *Staufen* in RNAi^73^. However, *Staufen*, which is found in other insect species has no role in this process^28^. The lower expression of *StauC* in RNAi resistant *L. decemlineata* cell lines (Lepd-SL1RR) showed processing of dsRNA to siRNA^28^. StauC domain is also missing in lepidopterans, which generally show poor RNAi response^74^. It may be possible that in other insect species, *Staufen* may be contributing to RNAi in place of *StauC*.

### dsRNA stability and improvement in RNAi efficiency through dsRNases knockdown

**Figure 12:**
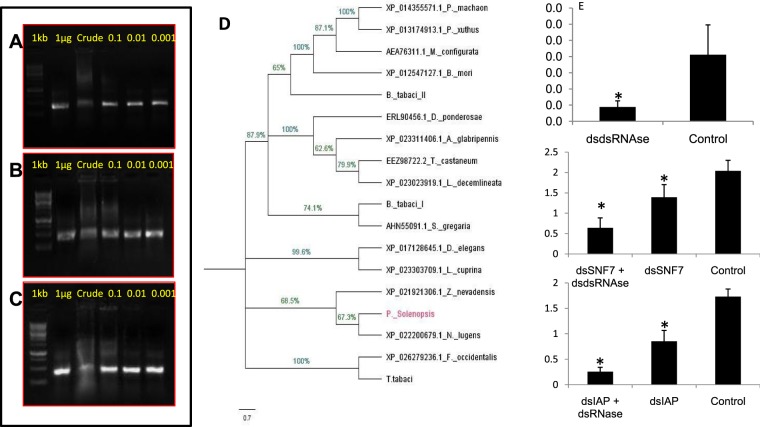
Stability of dsRNA in body juice at different time intervals analyzed through agarose gel electrophoresis. Stability of dsRNA after incubation in crude hemolymph and its serial dilutions of 0.1, 0.01, 0.001 for 1 hr (**A**), 3 hr (**B**), 5 hr (**C**). (**D**) Phylogenetic analysis of *dsRNAses* gene sequences across different insect species with MEGAX (5000 bootstrap value). Effect of consecutive knockdown of *dsRNAses* on RNAi of *SNF7* and *IAP* after injecting 10 µg dsRNA of each gene (**E**). *dsRNAses* knockdown (78.66%) (**F**). *SNF7* knockdown in *SNF7* + dsdsRNAse (68.5%) and *SNF7* (31.7%) as compared to control (**G**). *IAP* knockdown in *IAP* + dsdsRNAse (85.1%) and *IAP* (50.7%) as compared to control.

Hemipteran gut nucleases are the major bottlenecks in efficient RNAi resulting in degradation of dsRNA before it is processed inside the cell^75,76^. The serial dilutions of body fluids collected from *P. solenopsis* resulted in degradation of dsRNA at different time intervals. The concentration of fluid and time of incubation was directly proportional to the dsRNA degradation (Fig. 12). This clearly indicated that in polyphagous pest *P. solenopsis* gut, nucleases (possibly dsRNases) have a key role in hampering RNAi efficiency. The degradation of dsRNA in insect hemolymph and gut fluid has been demonstrated in many insect species^11^, *A. grandis*^63^, *Periplaneta americana*^77^ and *L. migratoria*^78^. Earlier studies on stink bug also suggest that *dsRNAse* activity is detrimental for RNAi in this insect^79^. The average RNAi knockdown efficiency irrespective of the gene in mealybug was about 62.53%, which indicates dsRNA injected against any gene will possibly cause approximately 60–65% reduction in mRNA transcripts of that target gene. Thus knockdown of *dsRNases* may result in at least 60–65% reduction in the *dsRNases* activity in the mealybug, despite degradation by this enzyme in the insect gut. However, actual knockdown percentage of *dsRNases* gene was 78.66%, which might have helped in protecting *IAP* and *SNF7* from degradation and resulted in enhancing the RNAi efficiency. The consecutive injection of dsRNase + dsSNF7 and dsRNase + dsIAP resulted in 68.5 and 85.1 per cent improved RNAi efficiency compared to dsGFP control, respectively (Fig. 12), while the injection of dsRNA alone against *SNF7* and *IAP* resulted in 31.7 and 50.7 per cent knockdown compared to dsGFP, respectively. Our previous studies with *B. tabaci*, supported that the knockdown of *dsRNases* resulted in improved RNAi efficiency of targeted genes^48^. Similar studies with *A. grandis* support that the knockdown of *dsRNases* resulted in enhanced RNAi of the targeted genes^63^. Thus studies on different isoforms of *dsRNases* and their interaction with dsRNA needs further attention in order to elucidate their complete role in RNAi and exploit these enzymes for improving the RNAi efficiency.

## Conclusions

The studies report the assembled and annotated, openly available transcriptome sequences resource of *P. solenopsis*, which carries information about genes involved in many biological processes besides RNAi in this insect. This sequence resource will provide valuable information to the researchers working or interested to work on this insect. The sequence resource has been useful in identifying and validating the existence of robust RNAi machinery in *P. solenopsis* through series of *in silico* and *in vivo* studies. The studies provide handful information on the scope of RNAi in one of the polyphagous crop pest, which can be employed for developing novel strategies for its management in future.

## Supplementary information

Supplementary information 1

Supplementary information 2

Supplementary information 3

SI_Fig.1, SI_Fig.2, SI_Fig.3, SI_Fig.4, SI_Table 1, SI_Table 2, SI_Table 3, SI_Table 4

## Data Availability

The data sets supporting the results of this article are deposited in National Center for Biotechnology Information (NCBI) repository under BioProject PRJNA436774, SRA (Sequence Read Archives) accession SRR6801044 and TSA (Transcriptome Shotgun Assembly) accession GGIT00000000. The Annotations, Gene ontology and KASS pathway analysis are included in MS-excel files in Supplementary information 1, Supplementary information 2 and Supplementary information 3, respectively.
