# Author Correction: Using *de novo* transcriptome assembly and analysis to study RNAi in *Phenacoccus solenopsis* Tinsley (Hemiptera: Pseudococcidae)

**DOI:** 10.1038/s41598-020-62488-9

**Published:** 2020-03-24

**Authors:** Satnam Singh, Mridula Gupta, Suneet Pandher, Gurmeet Kaur, Neha Goel, Pankaj Rathore

**Affiliations:** 1 Punjab Agricultural University, Regional Research Station, Faridkot, 151203 Punjab India; 2 Forest Research Institute, Dehradun, Uttaranchal India

Correction to: *Scientific Reports* 10.1038/s41598-019-49997-y, published online 23 September 2019

This Article contains errors.

Reference 17 was incorrectly given as:

Verma, M., Ghangal, R., Sharma, R., Sinha, A. K. & Jain, M. Transcriptome analysis of Catharanthus roseus for gene discovery and expression profiling. PLoS One. https://doi.org/10.1371/journal.pone.0103583 (2014).

The correct reference is listed below as ref. 1.

Additionally, a citation to reference 17 should be included in the Results and Discussion, where the sentence,

“Assembly of the available transcriptomes of P. solenopsis discovered the presence of 55198 transcripts in second instar (SRR6782025), 55569 transcripts in adult (SRR6782023), 49193 transcripts in third instar (SRR6782022) and 64497 in the eggs (SRR6782024)”

should read:

“Assembly of the available transcriptomes of P. solenopsis discovered the presence of 55198 transcripts in second instar (SRR6782025), 55569 transcripts in adult (SRR6782023), 49193 transcripts in third instar (SRR6782022) and 64497 in the eggs (SRR6782024)^17^”
