# Supplementary material for: Using de novo transcriptome assembly and analysis to study RNAi in Phenacoccus solenopsis Tinsley (Hemiptera: Pseudococcidae)
Source: Sci Rep. 2019 Sep 23;9:13710. doi: 10.1038/s41598-019-49997-y (PMC6757040; doi:10.1038/s41598-019-49997-y)
Supplement: Supplementary file 4 — SI_Fig.1, SI_Fig.2, SI_Fig.3, SI_Fig.4, SI_Table 1, SI_Table 2, SI_Table 3, SI_Table 4 [file 41598_2019_49997_MOESM4_ESM.pdf]

Using *de novo* transcriptome assembly and analysis to study RNAi in *Phenacoccus solenopsis* Tinsley (Hemiptera: Pseudococcidae)

Satnam Singh[1*], Mridula Gupta[1], Suneet Pandher[1], Gurmeet Kaur[1], Neha Goel[2], Pankaj Rathore[1]

[1] Punjab Agricultural University, Regional Station, Faridkot-151203, Punjab (India)

[2]Forest Research Institute, Dehradun, Uttaranchal (India)

* Corresponding author: satnam@pau.edu

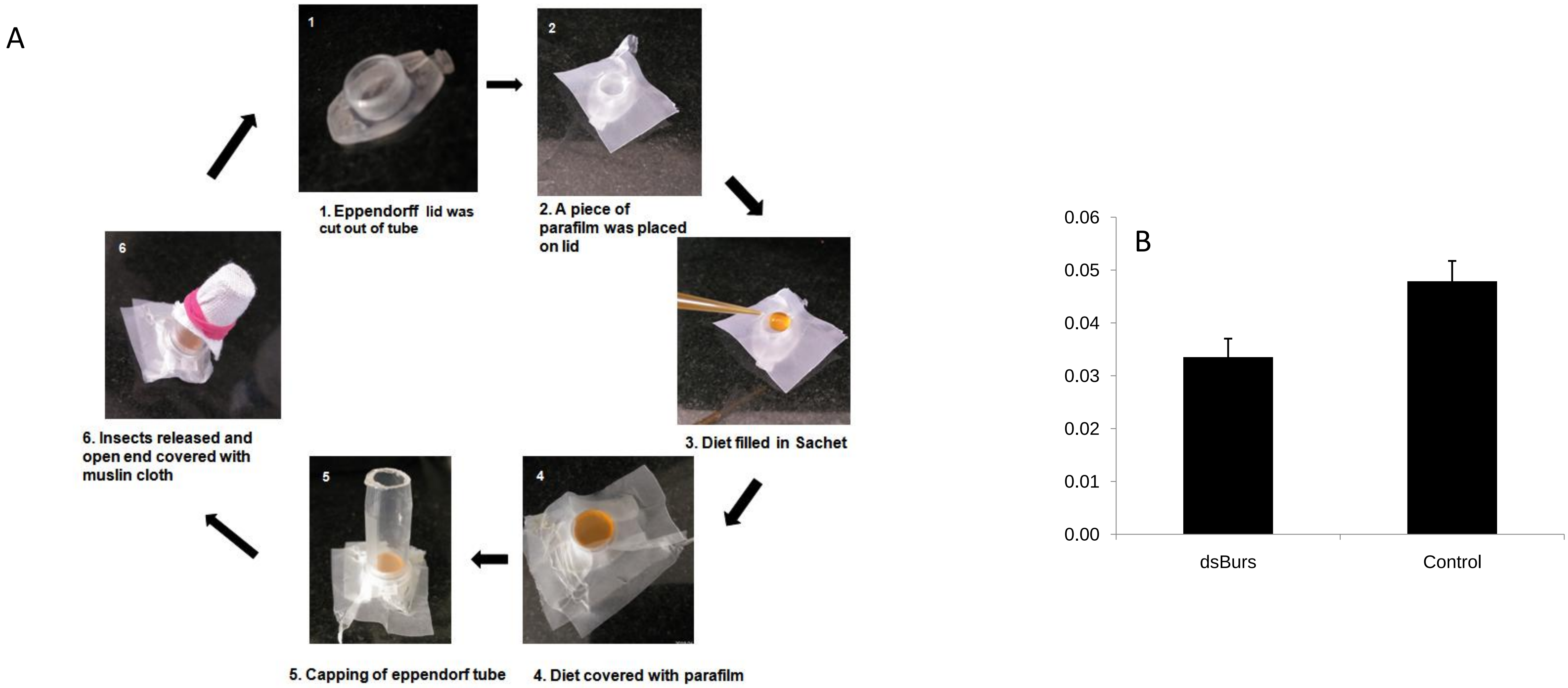

Supplementary Information 4 : SI_Fig. 1: A. Schematic diagram for membrane feeding of 2nd instar mealybug. B. Feeding dsBurs (dsRNA of Bursicon) in artificial diet to 2nd instar nymphs caused knockdown 30.01 % reduction in mRNA levels of *Bursicon* compared to control. The error bars represent the standard deviation (n=3) and the knockdown efficiency was non significant ($P \leq 0.05$, Student's t-test). Thus Petiole dip assay was preferred for feeding RNAi over membrane feeding assay.

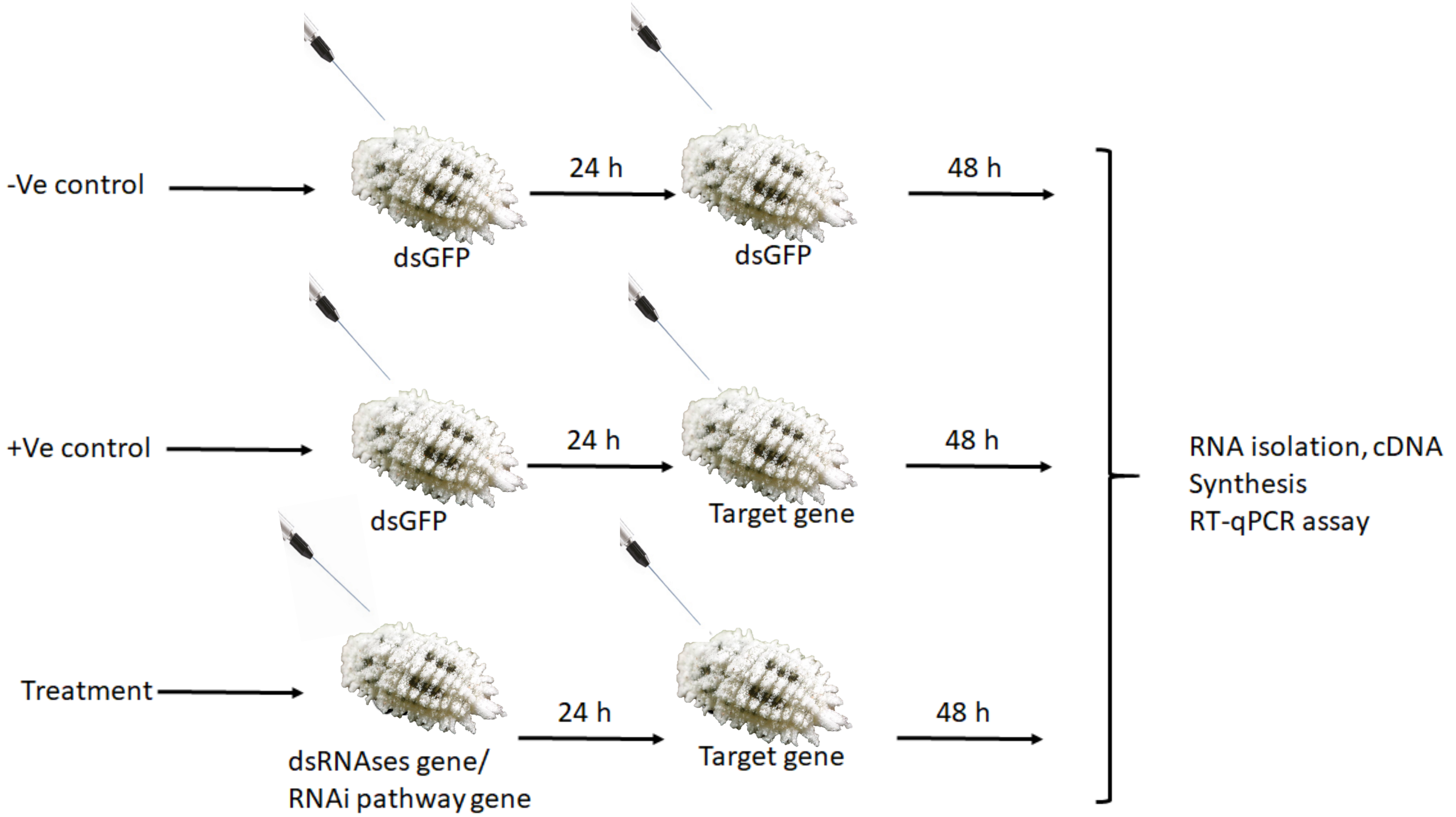

Supplementary Information 4 : SI_Fig.2 Schematic diagram for the methodology followed for assay of dsRNAses and RNAi pathway genes for studying their effect on knockdown efficiency of targeted gens

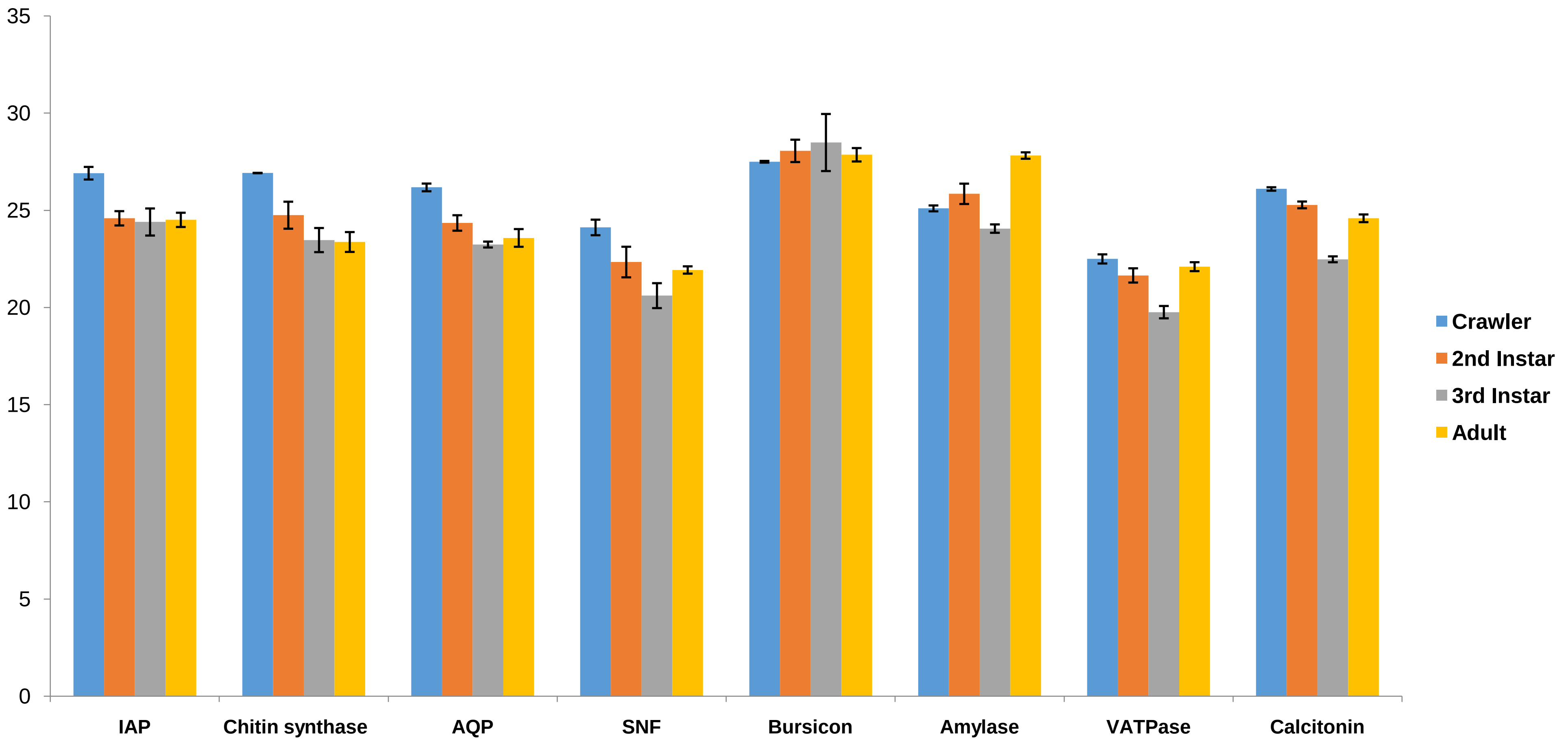

Supplementary information 4 SI_Fig. 3 Cq values±S.D. obtained for eight RNAi target genes in *P. solenopsis* across developmental stages. Each data point signifies the Mean±S. D. of Cq values for three biological replications in each developmental stage.

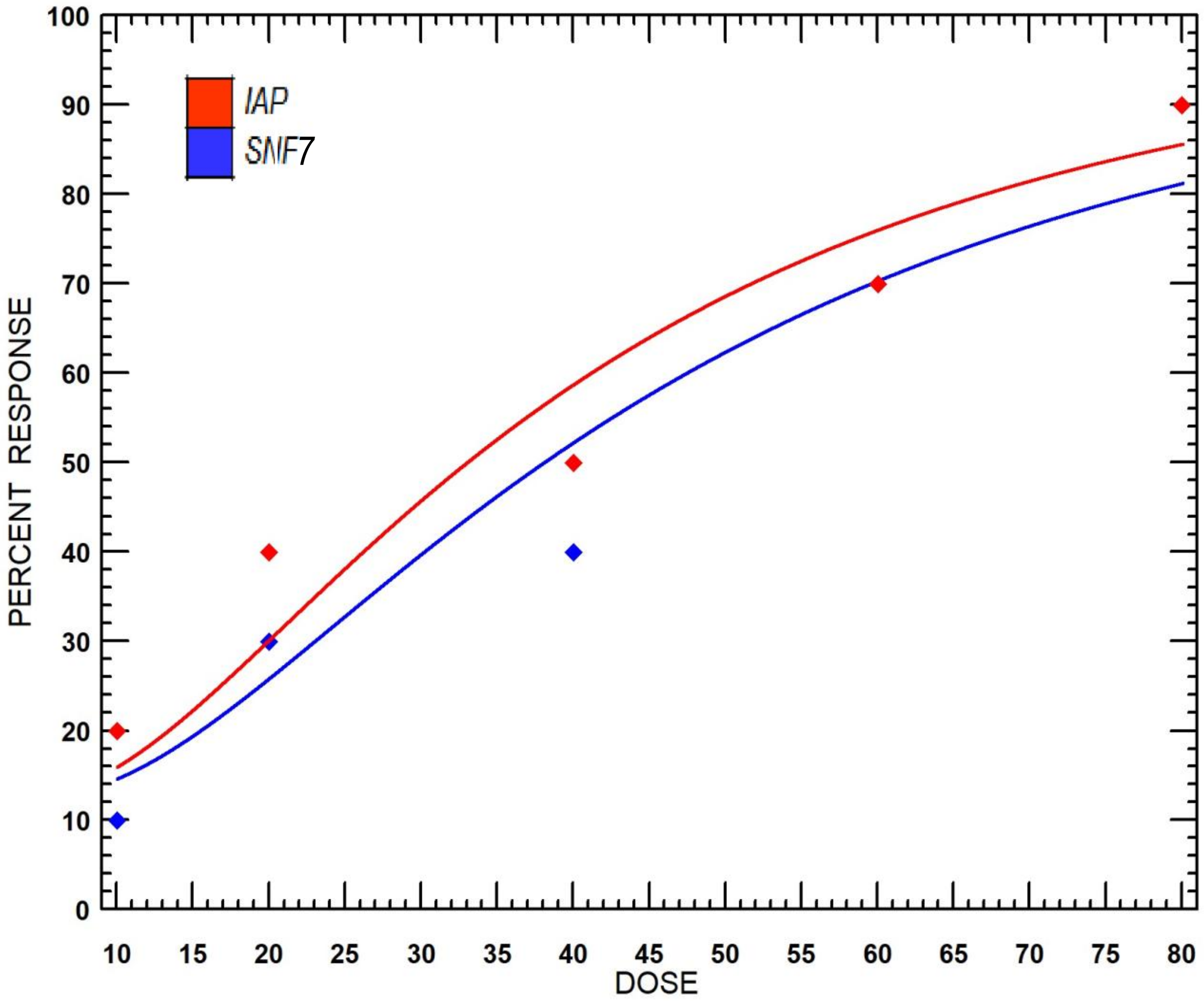

Supplementary information 4 SI_Fig. 4: Probit analysis to calculate mortality for dsSNF7 and dsIAP at different doses in insect

Supplementary Information SI_Table 1: Primer sequences of target genes of Phenacoccus solenopsis

Genes	Primers	Accession No.
Calcitonin_F	CCTATGGTTGGTATGGTACA	
Calcitonin_R	GTATATGTGGCTGCGTGCTATG	MK956911
IAP_F	GGTAGAGCATCGTCGTTATTC	
IAP_R	CGACCGAGTTGGCAGAATTA	MK956912
AQP_F	GGAATGATCTCGCCGATTAC	
AQP_R	TAGACGCTTCGCCCATATCT	MK956913
SNF7_F	GGGCCTTCCTTACCGAATAG	
SNF7_R	CATACGCCTGGTGTTTCTATCT	MK956916
Bursicon_F	GGGCCAATACCATCCTTTGC	
Bursicon_R	GCATTCTAGAGGTGCCTTTG	MK956917
Amylase_F	GCCTTGCCAGATGATTAC	
Amylase_R	GGTGTTCTAGCTTTGTCCCT	MK956914
VATPase_F	GCTCCTTATCCGTTCGGTATC	
VATPase_R	TCCGAGACCACTGTAGAATTTG	MH712873.1
ChitinSyntahse_F	GGGGTGGAGAGTGAAGATAC	
ChitinSyntahse_R	ACGACGTTCGGTTTGTAGAG3	MK956915

Supplementary information SI_Table 2: Transcripts involved in RNAi Machinery in *Phenacoccus solenopsis*

**Sequence name**	**Sequence description**	**Sequence length**	**Hit description**	**Hit Acession #**	**E-Value**	**Similarity**	**Alignment length**
Gene9128_Transcript_11147	argonaute partial	1350	gi|516306731|gb|AGO85968.1|argonaute 1, partial [Locusta migratoria]	AGO85968	0	99	411
Gene3146_Transcript_3574	protein argonaute-2 isoform x2	1089	gi|939687392|ref|XP_014287703.1|PREDICTED: protein argonaute-2 isoform X2 [Halyomorpha halys] >gi|939687396|ref|XP_014287704.1| PREDICTED: protein argonaute-2 isoform X2 [Halyomorpha halys]	XP_014287703	0	99	363
Gene3801_Transcript_4261	protein argonaute-2-like isoform x3	2670	gi|939641506|ref|XP_014271332.1|PREDICTED: protein argonaute-2-like isoform X3 [Halyomorpha halys]	XP_014271332	0	64	862
Gene42_Transcript_45	protein argonaute-2-like	534	gi|239791844|dbj|BAH72335.1|ACYPI008293 [Acyrthosiphon pisum]	BAH72335	1.1E-70	77	161
Gene8190_Transcript_9845	protein argonaute-2-like	639	gi|939240900|ref|XP_014239688.1|PREDICTED: protein argonaute-2-like [Cimex lectularius]	XP_014239688	1.2E-40	59	212
Gene9285_Transcript_11346	protein argonaute-2-like isoform x1	348	gi|939277460|ref|XP_014259236.1|PREDICTED: protein argonaute-2-like isoform X1 [Cimex lectularius] >gi|939277462|ref|XP_014259237.1| PREDICTED: protein argonaute-2-like isoform X1 [Cimex lectularius]	XP_014259236	6.3E-23	68	102
Gene10017_Transcript_12350	argonaute- partial	336	gi|426204656|gb|AFY13246.1|argonaute-2, partial [Schistocerca gregaria]	AFY13246	2.2E-10	52	118

Gene12903_Transcript_16707	protein argonaute-2	636	gi|645033016|ref|XP_008214880.1|PREDICTED: protein argonaute-2 [Nasonia vitripennis]	XP_008214880	2E-39	57	219
Gene15251_Transcript_21173	argonaute- partial	336	gi|426204656|gb|AFY13246.1|argonaute-2, partial [Schistocerca gregaria]	AFY13246	4E-10	52	118
Gene4726_Transcript_5389	endoribonuclease dicer	4296	gi|817086281|ref|XP_012265864.1|PREDICTED: endoribonuclease Dicer [Athalia rosae] >gi|817086283|ref|XP_012265865.1| PREDICTED: endoribonuclease Dicer [Athalia rosae]	XP_012265864	0	53	1489
Gene5260_Transcript_6138	dicer 2	4653	gi|406034947|emb|CCF23094.1|Dicer 2 [Blattella germanica]	CCF23094	0	51	1635
Gene6974_Transcript_8197	endoribonuclease dicer isoform x2	600	gi|939652846|ref|XP_014275311.1|PREDICTED: endoribonuclease Dicer isoform X2 [Halyomorpha halys]	XP_014275311	4.1E-40	64	183
Gene4616_Transcript_5250	dicer-1	1926	gi|240247227|emb|CAX68236.1|dicer-1 [Blattella germanica]	CAX68236	5E-164	58	715
Gene672_Transcript_675	dicer-1	1170	gi|411170363|gb|AFW16070.1|dicer-1 [Nilaparvata lugens]	AFW16070	3E-177	87	363

Gene1438_Transcript_1421	ribonuclease 3 isoform x2	2181	gi|936561856|ref|XP_014213264.1|PREDICTED: ribonuclease 3 isoform X2 [Copidosoma floridanum] >gi|936561859|ref|XP_014213272.1| PREDICTED: ribonuclease 3 isoform X2 [Copidosoma floridanum] >gi|936561862|ref|XP_014213280.1| PREDICTED: ribonuclease 3 isoform X2 [Copidosoma floridanum] >gi|936561865|ref|XP_014213290.1| PREDICTED: ribonuclease 3 isoform X2 [Copidosoma floridanum]	XP_014213264	0	85	707
Gene14701_Transcript_20015	ribonuclease 3	1419	gi|952511297|gb|KRT78218.1|hypothetical protein AMK59_8687, partial [Oryctes borbonicus]	KRT78218	3.1E-86	74	230
Gene4609_Transcript_5243	protein aubergine-like	2262	gi|516306739|gb|AGO85970.1|PIWI 2, partial [Locusta migratoria]	AGO85970	0	68	697
Gene5857_Transcript_6894	piwi-like protein 1	2760	gi|646703429|gb|KDR12118.1|Piwi-like protein 1 [Zootermopsis nevadensis]	KDR12118	0	65	832
Gene7823_Transcript_9404	clathrin heavy chain	5064	gi|566601502|gb|AHC70342.1|clathrin, partial [Locusta migratoria]	AHC70342	0	95	1654
Gene5995_Transcript_7047	epsin-2 isoform x1	552	gi|820848555|ref|XP_012342069.1|PREDICTED: epsin-2 [Apis florea]	XP_012342069	3.7E-11	52	122
Gene12975_Transcript_16803	epsin-2 isoform x3	519	gi|939638764|ref|XP_014270393.1|PREDICTED: epsin-1 isoform X6 [Halyomorpha halys]	XP_014270393	2E-100	91	173

Gene1868_ Transcript_ 1861	transferrin-like isoform x1	1248	gi|328714357|ref|XP_001946481 .2|PREDICTED: transferrin-like isoform X1 [Acyrthosiphon pisum] >gi|641667912|ref|XP_00818426 4.1| PREDICTED: transferrin-like isoform X1 [Acyrthosiphon pisum]	XP_001946481	6E-105	62	407
Gene12514 _Transcript _16084	PREDICTED: transferrin-like	1794	gi|817186381|ref|XP_012287691 .1|PREDICTED: transferrin-like [Orussus abietinus]	XP_012287691	7E-127	56	608
Gene1610_ Transcript_ 1586	scavenger receptor class b member 1 isoform x1	1605	gi|939679484|ref|XP_014285135 .1|PREDICTED: scavenger receptor class B member 1 isoform X1 [Halyomorpha halys]	XP_014285135	0	85	530
Gene5224_ Transcript_ 6092	scavenger receptor class b member 1	774	gi|662201869|ref|XP_008474202 .1|PREDICTED: scavenger receptor class B member 1-like [Diaphorina citri]	XP_008474202	9E-105	79	249
Gene7805_ Transcript_ 9386	scavenger receptor class b member 1-like isoform x2	1398	gi|985389068|ref|XP_015379122 .1|PREDICTED: scavenger receptor class B member 1-like isoform X2 [Diuraphis noxia]	XP_015379122	4E-160	74	390
Gene14106 _Transcript _18831	low-density lipoprotein receptor-related protein 2	2016	gi|328709384|ref|XP_001944217 .2|PREDICTED: low-density lipoprotein receptor-related protein 2 [Acyrthosiphon pisum]	XP_001944217	0	83	677
Gene14150 _Transcript _18913	low-density lipoprotein receptor-related protein 1	708	gi|985385516|ref|XP_015364616 .1|PREDICTED: low-density lipoprotein receptor-related protein 1 [Diuraphis noxia]	XP_015364616	2.9E-69	68	239
Gene14175 _Transcript _18984	low-density lipoprotein receptor-related protein 2- partial	615	gi|926640456|ref|XP_013786325 .1|PREDICTED: low-density lipoprotein receptor-related protein 2-like, partial [Limulus polyphemus]	XP_013786325	1.3E-38	58	205

Gene14222_Transcript_19058	low-density lipoprotein receptor-related protein 1	4425	gi|985385516|ref|XP_015364616.1|PREDICTED: low-density lipoprotein receptor-related protein 1 [Diuraphis noxia]	XP_015364616	0	58	1586
Gene3773_Transcript_4232	low-density lipoprotein receptor-related protein 2-like	2646	gi|926616750|ref|XP_013773508.1|PREDICTED: low-density lipoprotein receptor-related protein 2-like [Limulus polyphemus]	XP_013773508	4.2E-78	47	759
Gene4579_Transcript_5209	low-density lipoprotein receptor-related protein 5	1296	gi|915664608|gb|KOC66743.1|Low-density lipoprotein receptor-related protein 5 [Habropoda laboriosa]	KOC66743	5.8E-56	51	381
Gene4685_Transcript_5343	oxidized low-density lipoprotein receptor 1	792	gi|328713134|ref|XP_003245001.1|PREDICTED: oxidized low-density lipoprotein receptor 1 [Acyrthosiphon pisum]	XP_003245001	4.2E-95	76	260
Gene5181_Transcript_6033	low-density lipoprotein receptor-related protein 6	3540	gi|646695912|gb|KDR08547.1|Low-density lipoprotein receptor-related protein 6 [Zootermopsis nevadensis]	KDR08547	0	69	1109
Gene6387_Transcript_7509	low-density lipoprotein receptor-related protein 3-like	315	gi|926632472|ref|XP_013782004.1|PREDICTED: low-density lipoprotein receptor-related protein 3-like [Limulus polyphemus]	XP_013782004	4E-13	63	71
Gene9066_Transcript_11071	low-density lipoprotein receptor-related protein 2	2991	gi|646704521|gb|KDR12649.1|Low-density lipoprotein receptor-related protein 2 [Zootermopsis nevadensis]	KDR12649	0	79	1015
Gene1279_Transcript_1256	low-density lipoprotein receptor-related protein 2	6453	gi|328709384|ref|XP_001944217.2|PREDICTED: low-density lipoprotein receptor-related protein 2 [Acyrthosiphon pisum]	XP_001944217	0	88	2160

Gene1280_Transcript_1256	low-density lipoprotein receptor-related protein 2	1548	gi|985386327|ref|XP_015369125.1|PREDICTED: low-density lipoprotein receptor-related protein 2, partial [Diuraphis noxia]	XP_015369125	0	89	508
Gene3285_Transcript_3724	v-type proton atpase catalytic subunit a	852	gi|646720897|gb|KDR22470.1|V-type proton ATPase catalytic subunit A [Zootermopsis nevadensis]	KDR22470	0	95	284
Gene3445_Transcript_3894	v-type proton atpase subunit e	681	gi|121543569|gb|ABM55510.1|putative vacuolar ATP synthase subunit E [Maconellicoccus hirsutus]	ABM55510	2E-118	95	226
Gene3704_Transcript_4171	v-type proton atpase 116 kda subunit a isoform 1	492	gi|641675444|ref|XP_008187043.1|PREDICTED: V-type proton ATPase 116 kDa subunit a isoform 1 [Acyrthosiphon pisum]	XP_008187043	2.3E-51	80	161
Gene4446_Transcript_5046	v-type proton atpase 116 kda subunit a isoform 1- partial	528	gi|662205529|ref|XP_008476181.1|PREDICTED: V-type proton ATPase 116 kDa subunit a isoform 1-like, partial [Diaphorina citri]	XP_008476181	1.1E-93	91	176
Gene537_Transcript_549	v-type proton atpase 116 kda subunit a isoform 1 isoform x6	2025	gi|939679002|ref|XP_014284998.1|PREDICTED: V-type proton ATPase 116 kDa subunit a isoform 1 isoform X6 [Halyomorpha halys]	XP_014284998	0	87	675
Gene5614_Transcript_6639	v-type proton atpase catalytic subunit a	627	gi|239791896|dbj|BAH72354.1|ACYPI002584 [Acyrthosiphon pisum]	BAH72354	3E-142	97	208
Gene5619_Transcript_6645	v-type proton atpase subunit b	1506	gi|328716950|ref|XP_003246082.1|PREDICTED: V-type proton ATPase subunit B [Acyrthosiphon pisum]	XP_003246082	0	97	490

Gene6016_Transcript_7073	v-type proton atpase 116 kda subunit a isoform 1 isoform x3	1149	gi|985388666|ref|XP_015378903.1|PREDICTED: V-type proton ATPase 116 kDa subunit a isoform 1 isoform X3 [Diuraphis noxia]	XP_015378903	0	91	350
Gene6218_Transcript_7307	v-type proton atpase subunit c	1164	gi|193702420|ref|XP_001946227.1|PREDICTED: V-type proton ATPase subunit C [Acyrthosiphon pisum] >gi|641660018|ref|XP_008181338.1| PREDICTED: V-type proton ATPase subunit C [Acyrthosiphon pisum]	XP_001946227	0	94	387
Gene888_Transcript_894	v-type proton atpase 16 kda proteolipid subunit	456	gi|817207734|ref|XP_012279848.1|PREDICTED: V-type proton ATPase 16 kDa proteolipid subunit [Orussus abietinus]	XP_012279848	9.2E-60	92	151
Gene12393_Transcript_15904	v-type proton atpase 16 kda proteolipid subunit	474	gi|156550753|ref|XP_001600237.1|PREDICTED: V-type proton ATPase 16 kDa proteolipid subunit [Nasonia vitripennis]	XP_001600237	4.6E-93	99	154
Gene12512_Transcript_16082	v-type proton atpase 116 kda subunit a isoform 1-like	468	gi|808124622|ref|XP_012165459.1|PREDICTED: V-type proton ATPase 116 kDa subunit a isoform 1-like [Bombus terrestris]	XP_012165459	4.6E-62	86	127
Gene13948_Transcript_18554	v-type proton atpase subunit s1	789	gi|380024605|ref|XP_003696084.1|PREDICTED: uncharacterized protein LOC100865627 [Apis florea]	XP_003696084	2.4E-23	57	200
Gene4497_Transcript_5111	vacuolar atp synthase subunit	372	gi|665790581|ref|XP_008560982.1|PREDICTED: V-type proton ATPase subunit F 1 [Microplitis demolitor]	XP_008560982	8.8E-76	95	123
Gene8823_Transcript_10656	vacuolar atp synthase subunit d	630	gi|46561758|gb|AAT01084.1|putative vacuolar ATP synthase subunit D [Homalodisca vitripennis]	AAT01084	1E-140	99	207

Gene10316_Transcript_12796	vacuolar atp synthase 21 kda proteolipid subunit	423	gi|121543963|gb|ABM55646.1|putative vacuolar ATP synthase proteolipid subunit [Maconellicoccus hirsutus]	ABM55646	1.4E-60	87	143
Gene12919_Transcript_16729	vacuolar atpase g subunit-like protein	309	gi|121543630|gb|ABM55524.1|vacuolar ATPase G subunit-like protein [Maconellicoccus hirsutus]	ABM55524	3.9E-29	93	81
Gene13684_Transcript_18047	guanine nucleotide exchange factor for rab-3a-like isoform x3	798	gi|985410349|ref|XP_015370616.1|PREDICTED: guanine nucleotide exchange factor for Rab-3A-like isoform X3 [Diuraphis noxia]	XP_015370616	3.2E-65	81	166
Gene1587_Transcript_1559	rab gtpase-activating protein 1 isoform x1	2313	gi|646722305|gb|KDR23318.1|Rab GTPase-activating protein 1, partial [Zootermopsis nevadensis]	KDR23318	0	74	771
Gene15489_Transcript_21726	rab gtpase-activating protein 1 isoform x1	423	gi|646722305|gb|KDR23318.1|Rab GTPase-activating protein 1, partial [Zootermopsis nevadensis]	KDR23318	3.3E-54	82	130
Gene4452_Transcript_5049	rab6 gtpase activating	312	gi|242017802|ref|XP_002429375.1|rab6 GTPase activating protein, gapcena, putative [Pediculus humanus corporis] >gi|212514288|gb|EEB16637.1|rab6 GTPase activating protein, gapcena, putative [Pediculus humanus corporis]	XP_002429375	5.7E-07	65	64
Gene6169_Transcript_7260	rab gtpase-binding effector protein 1 isoform x3	1227	gi|646688535|gb|KDR06357.1|hypothetical protein L798_04283, partial [Zootermopsis nevadensis]	KDR06357	2E-112	73	349
Gene7787_Transcript_9363	rab3 gtpase-activating protein catalytic subunit	2076	gi|642923339|ref|XP_008193708.1|PREDICTED: rab3 GTPase-activating protein catalytic subunit [Tribolium castaneum]	XP_008193708	5E-147	58	707

Gene781_Transcript_789	rab3 gtpase-activating protein catalytic subunit	633	gi|662201644|ref|XP_008474082.1|PREDICTED: LOW QUALITY PROTEIN: uncharacterized protein LOC103511145 [Diaphorina citri]	XP_008474082	9.6E-27	56	205
Gene9672_Transcript_11823	rab3 gtpase-activating protein non-catalytic subunit	4017	gi|936572042|ref|XP_014218422.1|PREDICTED: rab3 GTPase-activating protein regulatory subunit isoform X1 [Copidosoma floridanum]	XP_014218422	0	55	1399
Gene1083_Transcript_1078	adp-ribosylation arf	420	gi|158300992|ref|XP_320779.3|AGAP011730-PA [Anopheles gambiae str. PEST] >gi|157013426|gb|EAA00052.3|AGAP011730-PA [Anopheles gambiae str. PEST]	XP_320779	8E-76	94	139
Gene10965_Transcript_13743	adp-ribosylation factor-like protein 13b	1245	gi|646707352|gb|KDR14158.1|ADP-ribosylation factor-like protein 13B [Zootermopsis nevadensis]	KDR14158	1.2E-66	66	277
Gene1105_Transcript_1097	adp ribosylation factor 102f	570	gi|568255707|gb|ETN64433.1|ADP ribosylation factor 102F [Anopheles darlingi] >gi|668457326|gb|KFB45367.1|hypothetical protein ZHAS_00013319 [Anopheles sinensis]	ETN64433	2E-116	96	179

Gene14263_Transcript_19125	adp-ribosylation factor-related protein 1	606	gi|[illegible]|ref|XP_011687533.1|PREDICTED: ADP-ribosylation factor-related protein 1 [Wasmannia auropunctata] >gi|815795692|ref|XP_012218502.1| PREDICTED: ADP-ribosylation factor-related protein 1 isoform X1 [Linepithema humile] >gi|826412849|ref|XP_012543513.1| PREDICTED: ADP-ribosylation factor-related protein 1 [Monomorium pharaonis]	XP_011687533	1E-120	92	199
Gene14501_Transcript_19600	adp-ribosylation factor-like protein 3 isoform x2	543	gi|193580240|ref|XP_001949993.1|PREDICTED: ADP-ribosylation factor-like protein 3 isoform X2 [Acyrthosiphon pisum] >gi|985384640|ref|XP_015365815.1| PREDICTED: ADP-ribosylation factor-like protein 3 isoform X2 [Diuraphis noxia]	XP_001949993	1E-104	91	180
Gene3480_Transcript_3927	adp-ribosylation factor-like protein 2	579	gi|91087357|ref|XP_975625.1|PREDICTED: ADP-ribosylation factor-like protein 2 [Tribolium castaneum] >gi|270010616|gb|EFA07064.1| hypothetical protein TcasGA2_TC010041 [Tribolium castaneum]	XP_975625	4.6E-83	91	160

Gene5740_Transcript_6776	adp-ribosylation factor-like protein 6-interacting protein 4	339	gi|662188172|ref|XP_008487746.1|PREDICTED: ADP-ribosylation factor-like protein 6-interacting protein 4 [Diaphorina citri] >gi|662188174|ref|XP_008487747.1| PREDICTED: ADP-ribosylation factor-like protein 6-interacting protein 4 [Diaphorina citri]	XP_008487746	1E-06	77	40
Gene6130_Transcript_7219	adp-ribosylation factor-like protein 5b isoform x1	540	gi|817071423|ref|XP_012257776.1|PREDICTED: ADP-ribosylation factor-like protein 5B isoform X1 [Athalia rosae]	XP_012257776	3E-113	93	179
Gene6914_Transcript_8124	adp-ribosylation factor-binding protein gga3	1923	gi|972211465|ref|XP_015188148.1|PREDICTED: ADP-ribosylation factor-binding protein GGA3 [Polistes dominula]	XP_015188148	1E-102	55	687
Gene8021_Transcript_9639	adp-ribosylation factor gtpase-activating	1623	gi|985397038|ref|XP_015366044.1|PREDICTED: ADP-ribosylation factor GTPase-activating protein 2 [Diuraphis noxia]	XP_015366044	5E-156	67	549

Gene8201_ Transcript_ 9860	adp-ribosylation factor 6	528	.1|PREDICTED: ADP-ribosylation factor 6 [Bombus terrestris] >gi|345498024|ref|XP_003428126.1| PREDICTED: ADP-ribosylation factor 6 [Nasonia vitripennis] >gi|345498026|ref|XP_001603804.2| PREDICTED: ADP-ribosylation factor 6 [Nasonia vitripennis] >gi|350406774|ref|XP_003487878.1| PREDICTED: ADP-ribosylation factor 6 isoform X1 [Bombus impatiens] >gi|380019019|ref|XP_003693415.1| PREDICTED: ADP-ribosylation factor 6 [Apis florea] >gi|383847271|ref|XP_003699278.1| PREDICTED: ADP-ribosylation factor 6 isoform X2 [Megachile rotundata] >gi|512922083|ref|XP_004930032.1| PREDICTED: ADP-ribosylation factor 6 [Bombyx mori] >gi|572307215|ref|XP_006619479.1| PREDICTED: ADP-ribosylation factor 6-like [Apis dorsata]	XP_003399058	4E-125	100	175
Gene8451_ Transcript_ 10169	adp-ribosylation factor-like protein 4c	621	gi|914562894|gb|KOB68938.1|ADP-ribosylation factor-like protein 4A [Operophtera brumata]	KOB68938	5E-117	91	195

Gene9039_Transcript_11027	adp-ribosylation factor 1	429	gi|939648957|ref|XP_014273936.1|PREDICTED: ADP-ribosylation factor 1 [Halyomorpha halys] >gi|939648959|ref|XP_014273937.1| PREDICTED: ADP-ribosylation factor 1 [Halyomorpha halys]	XP_014273936	3.9E-98	100	143
Gene9063_Transcript_11064	adp-ribosylation factor gtpase-activating protein 1 isoform x1	1086	gi|939233759|ref|XP_014241811.1|PREDICTED: ADP-ribosylation factor GTPase-activating protein 1 isoform X1 [Cimex lectularius]	XP_014241811	1.3E-71	64	282
Gene10371_Transcript_12869	adp-ribosylation factor-like protein 8b-a	336	gi|328712016|ref|XP_003244709.1|PREDICTED: ADP-ribosylation factor-like protein 8B-A [Acyrthosiphon pisum] >gi|985403789|ref|XP_015369611.1| PREDICTED: ADP-ribosylation factor-like protein 8B-A [Diuraphis noxia]	XP_003244709	3.6E-67	96	108
Gene7779_Transcript_9354	small rna degrading nuclease 5 isoform x1	1917	gi|817073780|ref|XP_012259049.1|PREDICTED: small RNA degrading nuclease 5 isoform X2 [Athalia rosae]	XP_012259049	2E-145	63	587
Gene1727_Transcript_1715	hermansky-pudlak syndrome 3 protein homolog isoform x1	2661	gi|939676728|ref|XP_014284227.1|PREDICTED: Hermansky-Pudlak syndrome 3 protein homolog isoform X2 [Halyomorpha halys]	XP_014284227	3.6E-41	42	951

Gene4502_Transcript_5120	hermansky-pudlak syndrome 1 protein homolog	1350	gi|328707951|ref|XP_001947892.2|PREDICTED: Hermansky-Pudlak syndrome 1 protein homolog [Acyrthosiphon pisum] >gi|641660668|ref|XP_008181570.1| PREDICTED: Hermansky-Pudlak syndrome 1 protein homolog [Acyrthosiphon pisum]	XP_001947892	1.9E-71	57	434
Gene6833_Transcript_8046	hermansky-pudlak syndrome 4 protein homolog isoform x2	2298	gi|939238527|ref|XP_014261955.1|PREDICTED: uncharacterized protein LOC106674035 isoform X1 [Cimex lectularius]	XP_014261955	4E-142	53	780
Gene9133_Transcript_11156	hermansky-pudlak syndrome 1 protein	549	gi|646701961|gb|KDR11428.1|Hermansky-Pudlak syndrome 1 protein-like protein [Zootermopsis nevadensis]	KDR11428	2.6E-76	79	179
Gene3307_Transcript_3754	dead box atp-dependent rna helicase-like partial	606	gi|506968895|gb|AGM32791.1|DEAD box ATP-dependent RNA helicase-like protein, partial [Coptotermes formosanus]	AGM32791	3E-110	91	201
Gene5469_Transcript_6476	dead box atp-dependent rna helicase	378	gi|170071047|ref|XP_001869798.1|DEAD box ATP-dependent RNA helicase [Culex quinquefasciatus] >gi|167866996|gb|EDS30379.1| DEAD box ATP-dependent RNA helicase [Culex quinquefasciatus]	XP_001869798	3.4E-84	99	125
Gene10557_Transcript_13106	dead box atp-dependent rna	2403	gi|328723280|ref|XP_001943847.2|PREDICTED: probable ATP-dependent RNA helicase DDX20 [Acyrthosiphon pisum]	XP_001943847	5.9E-89	62	406

Gene11894_Transcript_15117	dead-box helicase dbp80	1002	gi|646714912|gb|KDR18709.1|DEAD-box helicase Dbp80 [Zootermopsis nevadensis]	KDR18709	8E-135	86	258
Gene1351_Transcript_1319	dead box atp-dependent rna helicase	2088	gi|501295923|dbj|BAN20948.1|DEAD box ATP-dependent RNA helicase [Riptortus pedestris]	BAN20948	0	85	423
Gene13970	dead box atp-	645	EAD box ATP-dependent RNA	AGM32791	2E-109	91	201
Gene2362_Transcript_2432	protein abnormal spindle	1107	gi|646720534|gb|KDR22214.1|Protein abnormal spindle [Zootermopsis nevadensis]	KDR22214	1.6E-24	46	365
Gene2900_Transcript_3160	protein abnormal spindle-like isoform x2	1677	gi|985409495|ref|XP_015370146.1|PREDICTED: protein abnormal spindle-like isoform X2 [Diuraphis noxia]	XP_015370146	1.2E-21	42	635
Gene8995_Transcript_10955	protein abnormal spindle	573	gi|91080169|ref|XP_970176.1|PREDICTED: protein abnormal spindle [Tribolium castaneum]	XP_970176	6.9E-24	61	192
Gene9359_Transcript_11438	spindle assembly abnormal protein 6 homolog	1212	gi|817212012|ref|XP_012282172.1|PREDICTED: spindle assembly abnormal protein 6 homolog [Orussus abietinus]	XP_012282172	2.4E-20	51	337
Gene15684_Transcript_22192	spindle assembly abnormal protein 6-like protein	462	gi|646715514|gb|KDR19091.1|Spindle assembly abnormal protein 6-like protein [Zootermopsis nevadensis]	KDR19091	4.6E-15	52	149
Gene9648_Transcript_11795	rna binding protein fox-1 homolog 2-like isoform x7	1059	gi|951517270|ref|XP_014489079.1|PREDICTED: RNA binding protein fox-1 homolog 2-like isoform X7 [Dinoponera quadriceps]	XP_014489079	2.6E-42	71	126
Gene11782_Transcript_14945	rna binding protein	339	gi|952530824|gb|KRT83885.1|RNA binding protein [Oryctes borbonicus]	KRT83885	2.2E-25	96	57
Gene15039_Transcript_20698	rna binding protein	366	gi|478251698|gb|ENN72152.1|hypothetical protein YQE_11209, partial [Dendroctonus ponderosae]	ENN72152	3.5E-53	94	108

Gene3139_Transcript_3564	rna binding partial	594	gi|952512384|gb|KRT79126.1|RNA binding protein, partial [Oryctes borbonicus]	KRT79126	9E-110	94	176
Gene6274_Transcript_7368	rna binding motif protein x-linked 2	420	gi|357611289|gb|EHJ67406.1|RNA binding motif protein X-linked 2 [Danaus plexippus]	EHJ67406	8.4E-58	88	107
Gene15449_Transcript_21644	protein arginine n-methyltransferase 1	342	gi|972206976|ref|XP_015185745.1|PREDICTED: protein arginine N-methyltransferase 1 [Polistes dominula]	XP_015185745	1.8E-34	73	109
Gene3626_Transcript_4082	protein arginine n-methyltransferase 8	1107	gi|646689677|gb|KDR06666.1|Protein arginine N-methyltransferase 8 [Zootermopsis nevadensis]	KDR06666	0	87	358
Gene4217_Transcript_4763	protein arginine n-methyltransferase 5	1869	gi|646718675|gb|KDR21061.1|Protein arginine N-methyltransferase 5 [Zootermopsis nevadensis]	KDR21061	0	69	623
Gene5341_Transcript_6273	protein arginine n-methyltransferase 9	2142	gi|939684922|ref|XP_014286911.1|PREDICTED: putative protein arginine N-methyltransferase 9 [Halyomorpha halys]	XP_014286911	4E-90	52	709
Gene6224_Transcript_7315	protein arginine n-methyltransferase 1	600	gi|26353886|dbj|BAC40573.1|unnamed protein product [Mus musculus]	BAC40573	1E-33	58	162
Gene8949_Transcript_10875	protein arginine n-methyltransferase 7	1953	gi|646710226|gb|KDR15803.1|Protein arginine N-methyltransferase 7 [Zootermopsis nevadensis]	KDR15803	0	67	653
Gene9751_Transcript_11925	protein arginine n-methyltransferase 6	1089	gi|642927466|ref|XP_008195284.1|PREDICTED: protein arginine N-methyltransferase 6 [Tribolium castaneum]	XP_008195284	8E-101	67	337

Gene10397_Transcript_12911	protein arginine n-methyltransferase 1	1023	gi|939279237|ref|XP_014260190.1|PREDICTED: protein arginine N-methyltransferase 1 [Cimex lectularius] >gi|939279239|ref|XP_014260191.1| PREDICTED: protein arginine N-methyltransferase 1 [Cimex lectularius]	XP_014260190	4E-106	69	323
Gene10512_Transcript_13063	protein arginine n-	687	gi|242014396|ref|XP_002427877.1|protein arginine N-methyltransferase, putative [Pediculus humanus corporis] >gi|212512346|gb|EEB15139.1| protein arginine N-methyltransferase, putative [Pediculus humanus corporis]	XP_002427877	2.3E-15	51	224
Gene12394_Transcript_15906	maternal protein tudor-like isoform x2	906	gi|826478596|ref|XP_012537619.1|PREDICTED: maternal protein tudor-like isoform X2 [Monomorium pharaonis]	XP_012537619	1.1E-25	54	235
Gene1274_Transcript_1249	tudor domain-containing protein 7	2298	gi|409194603|gb|AFV31612.1|tdrd7 [Gryllus bimaculatus]	AFV31612	1.3E-65	50	580
Gene14706_Transcript_20031	tudor domain-containing protein 12	723	gi|646710823|gb|KDR16241.1|Tudor domain-containing protein 12 [Zootermopsis nevadensis]	KDR16241	5.7E-17	55	141

Gene14745_Transcript_20105	tudor domain-containing protein 7b-like isoform x1	615	gi|817217546|ref|XP_012284701.1|PREDICTED: tudor domain-containing protein 7A [Orussus abietinus] >gi|817217549|ref|XP_012284703.1| PREDICTED: tudor domain-containing protein 7A [Orussus abietinus] >gi|817217551|ref|XP_012284704.1| PREDICTED: tudor domain-containing protein 7A [Orussus abietinus] >gi|817217554|ref|XP_012284705.1| PREDICTED: tudor domain-containing protein 7A [Orussus abietinus]	XP_012284701	1.9E-13	49	202
Gene15609_Transcript_22006	tudor domain-containing protein 1	576	gi|646715533|gb|KDR19110.1|Tudor domain-containing protein 1 [Zootermopsis nevadensis]	KDR19110	6.8E-15	48	189
Gene1960_Transcript_1963	tudor domain-containing protein 1 isoform x1	1632	gi|939649280|ref|XP_014274051.1|PREDICTED: uncharacterized protein LOC106679416 [Halyomorpha halys]	XP_014274051	1.3E-08	65	73
Gene3006_Transcript_3347	tudor domain-containing protein 7-like isoform x1	372	gi|157125859|ref|XP_001654423.1|AAEL010311-PA [Aedes aegypti] >gi|108873488|gb|EAT37713.1|AAEL010311-PA [Aedes aegypti]	XP_001654423	1.4E-10	52	128
Gene3369_Transcript_3817	tudor domain-containing protein 1 isoform x1	1242	gi|939649280|ref|XP_014274051.1|PREDICTED: uncharacterized protein LOC106679416 [Halyomorpha halys]	XP_014274051	3.9E-21	44	376

Gene3816_Transcript_4277	tudor domain-containing protein 1-like	333	gi|926641110|ref|XP_013786678.1|PREDICTED: uncharacterized protein LOC106470659, partial [Limulus polyphemus]	XP_013786678	2.1E-11	50	112
Gene4769_Transcript_5450	tudor domain-containing protein 5	3156	gi|646703152|gb|KDR11968.1|Tudor domain-containing protein 5 [Zootermopsis nevadensis]	KDR11968	1.2E-32	44	522
Gene774_Transcript_782	maternal protein tudor isoform x1	627	gi|815921522|ref|XP_012245640.1|PREDICTED: maternal protein tudor isoform X3 [Bombus impatiens]	XP_012245640	2.5E-11	51	172
Gene7853_Transcript_9434	tudor domain-containing protein 7a-like	723	gi|817073456|ref|XP_012258869.1|PREDICTED: tudor domain-containing protein 7A-like isoform X2 [Athalia rosae]	XP_012258869	7.6E-15	66	90
Gene9746_Transcript_11912	tudor domain-containing protein 7-like isoform x2	615	gi|157125859|ref|XP_001654423.1|AAEL010311-PA [Aedes aegypti] >gi|108873488|gb|EAT37713.1|AAEL010311-PA [Aedes aegypti]	XP_001654423	3.2E-31	56	205
Gene986_Transcript_989	tudor and kh domain-containing	1200	gi|662213226|ref|XP_008480390.1|PREDICTED: tudor and KH domain-containing protein-like [Diaphorina citri]	XP_008480390	1.4E-41	49	364
Gene10660_Transcript_13254	tudor domain-containing protein 1	2271	gi|646715533|gb|KDR19110.1|Tudor domain-containing protein 1 [Zootermopsis nevadensis]	KDR19110	1.1E-16	42	349
Gene13540_Transcript_17773	atp-dependent clp protease atp-binding subunit clpx-mitochondrial isoform x1	1836	gi|646712236|gb|KDR17084.1|ATP-dependent Clp protease ATP-binding subunit clpX-like, mitochondrial [Zootermopsis nevadensis]	KDR17084	0	87	505

Gene12427_Transcript_15966	atp-dependent clp protease proteolytic mitochondrial	717	gi|646720905|gb|KDR22478.1|Putative ATP-dependent Clp protease proteolytic subunit, mitochondrial [Zootermopsis nevadensis]	KDR22478	1E-127	91	212
Gene2042_Transcript_2064	reverse rna-dependent dna polymerase domain-containing protein	507	gi|528890629|gb|EPZ31199.1|Reverse transcriptase, RNA-dependent DNA polymerase domain-containing protein [Rozella allomycis CSF55]	EPZ31199	3.4E-07	58	75
Gene4609_Transcript_5243	protein aubergine-like	2262	gi|516306739|gb|AGO85970.1|PIWI 2, partial [Locusta migratoria]	AGO85970	0	68	697
Gene1983_Transcript_1997	risc-loading complex subunit tarbp2-like isoform x1	978	gi|951535376|ref|XP_014471217.1|PREDICTED: RISC-loading complex subunit TARBP2-like isoform X1 [Dinoponera quadriceps]	XP_014471217	1.8E-20	45	341

Supplementary information SI_Table 3: Transcripts involved in RNAi Machinery in Phenacoccus solenopsis

**Sequence name**	**Sequence description**	**Sequence length**	**Hit descrption**	**Hit Acession #**	**E-Value**	**Similarity**	**Alignment length**
Gene10997_Transcript_13810	vacuolar h	1380	gi|501293539|dbj|BAN20615.1|vacuolar H[] ATPase subunit [Riptortus pedestris]	BAN20615	0	89	461
Gene12538_Transcript_16116	tubulin-folding cofactor b	762	gi|913324565|ref|XP_013193607.1|PREDICTED: tubulin-folding cofactor B [Amyelois transitella]	XP_013193607	6.62605E-60	64	252
Gene12514_Transcript_16084	PREDICTED: transferrin-like	1794	gi|817186381|ref|XP_012287691.1|PREDICTED: transferrin-like [Orussus abietinus]	XP_012287691	6.5393E-127	56	608
Gene6001_Transcript_7054	glutamyl-trna amidotransferase subunit mitochondrial	1488	gi|646699587|gb|KDR10141.1|Glutamyl-tRNA(Gln) amidotransferase subunit A-like protein [Zootermopsis nevadensis]	KDR10141	6.3114E-172	70	503
Gene617_Transcript_627	28s ribosomal protein mitochondrial-like	501	gi|913302294|ref|XP_013200341.1|PREDICTED: 28S ribosomal protein S2, mitochondrial-like [Amyelois transitella]	XP_013200341	2.26408E-45	70	148
Gene8506_Transcript_10241	elongation of very long chain fatty acids protein aael008004-like isoform x2	822	gi|985399759|ref|XP_015367517.1|PREDICTED: elongation of very long chain fatty acids protein AAEL008004-like [Diuraphis noxia]	XP_015367517	2.4072E-157	92	264
Gene9672_Transcript_11823	rab3 gtpase-activating protein non-catalytic subunit	4017	gi|936572042|ref|XP_014218422.1|PREDICTED: rab3 GTPase-activating protein regulatory subunit isoform X1 [Copidosoma floridanum]	XP_014218422	0	55	1399

Gene10258_Transcript_12711	aquaporin	744	gi|208609314|dbj|BAG72254.1|aquaporin [Coptotermes formosanus]	BAG72254	2.82858E-93	72	243
Gene11098_Transcript_13966	cathepsin b-like cysteine proteinase 4	672	gi|985396557|ref|XP_015365780.1|PREDICTED: cathepsin B-like cysteine proteinase 4 [Diuraphis noxia]	XP_015365780	7.00674E-66	61	239
Gene15042_Transcript_20711	golgi snap receptor complex member 1	708	gi|646717139|gb|KDR20120.1|Golgi SNAP receptor complex member 1 [Zootermopsis nevadensis]	KDR20120	3.6821E-109	83	234
Gene12640_Transcript_16263	actin-related protein	1080	gi|501297067|dbj|BAN21097.1|actin-related protein [Riptortus pedestris]	BAN21097	0	94	358
Gene13947_Transcript_18552	adp atp translocase	930	gi|478346160|gb|AGI96985.1|ADP/ATP translocase [Nilaparvata lugens] >gi|787071538|dbj|BAR13242.1|adenine nucleotide translocase insect1 [Nilaparvata lugens]	AGI96985	0	95	300
Gene7807_Transcript_9388	protein toll	891	gi|646706355|gb|KDR13629.1|Protein toll [Zootermopsis nevadensis]	KDR13629	2.07135E-25	55	232
Gene3468_Transcript_3918	vitellogenin isoform x1	1332	gi|985397648|ref|XP_015366382.1|PREDICTED: vitellogenin isoform X1 [Diuraphis noxia]	XP_015366382	5.82E-110	63	437
Gene12751_Transcript_16434	protein white-like	1023	gi|662190368|ref|XP_008467946.1|PREDICTED: uncharacterized protein LOC103505393 [Diaphorina citri]	XP_008467946	7.4934E-121	83	280

Gene2226_Transcript_2284	carboxylesterase	1641	gi|586831179|gb|AHJ81323.1|carboxylesterase [Locusta migratoria]	AHJ81323	1.90702E-71	51	556
Gene2609_Transcript_2758	cytochrome p450 6bq11	1566	gi|646716643|gb|KDR19800.1|putative cytochrome P450 6a13 [Zootermopsis nevadensis]	KDR19800	6.5181E-115	55	511
Gene5961_Transcript_7013	acetylcholinesterase 1	2625	gi|985414258|ref|XP_015372572.1|PREDICTED: acetylcholinesterase 1 [Diuraphis noxia]	XP_015372572	0	74	744
Gene12172_Transcript_15491	glutathione s-transferase c-terminal domain-containing protein	1365	gi|646716168|gb|KDR19528.1|Glutathione S-transferase C-terminal domain-containing protein-like protein [Zootermopsis nevadensis]	KDR19528	8.5231E-138	63	475
Gene170_Transcript_184	inter-alpha-trypsin inhibitor heavy chain h3 isoform x2	783	gi|328712314|ref|XP_001943110.2|PREDICTED: inter-alpha-trypsin inhibitor heavy chain H3 isoform X2 [Acyrthosiphon pisum]	XP_001943110	1.23378E-80	75	245
Gene13116_Transcript_17002	coagulation factor x	960	gi|939279779|ref|XP_014260482.1|PREDICTED: chymotrypsinogen A-like [Cimex lectularius]	XP_014260482	2.36912E-24	50	254
Gene10279_Transcript_12750	juvenile hormone acid methyltransferase	819	gi|631793773|gb|AHZ20738.1|juvenile hormone acid methyltransferase [Diploptera punctata]	AHZ20738	3.5274E-58	55	273
Gene11975_Transcript_15219	homeodomain transcription factor	876	gi|646708434|gb|KDR14746.1|Putative homeodomain transcription factor [Zootermopsis nevadensis]	KDR14746	1.56701E-27	68	141

Gene3355_Transcript_3808	chitin deacetylase 2	816	gi|762060959|gb|AJQ20733.1|chitin deacetylase 2 [Nilaparvata lugens]	AJQ20733	2.0716E-161	86	277
Gene5817_Transcript_6847	probable chitinase 3	615	gi|939264426|ref|XP_014252214.1|PREDICTED: probable chitinase 3 [Cimex lectularius]	XP_014252214	6.7854E-128	91	204
Gene6609_Transcript_7763	carboxypeptidase d	2937	gi|985400959|ref|XP_015368174.1|PREDICTED: carboxypeptidase D [Diuraphis noxia]	XP_015368174	0	58	919
Gene6594_Transcript_7745	atpase n2b	1416	gi|646713527|gb|KDR17842.1|Putative ATPase N2B [Zootermopsis nevadensis]	KDR17842	0	79	410
Gene3458_Transcript_3908	cullin-1 isoform x2	2310	gi|646702041|gb|KDR11477.1|Cullin-1 [Zootermopsis nevadensis]	KDR11477	0	91	768
Gene3569_Transcript_4021	homeobox protein homothorax isoform x3	1227	gi|939663689|ref|XP_014279414.1|PREDICTED: homeobox protein homothorax isoform X3 [Halyomorpha halys]	XP_014279414	0	85	410
Gene12025_Transcript_15291	pumilio homolog 2 isoform x7	2214	gi|939249020|ref|XP_014244019.1|PREDICTED: pumilio homolog 2 isoform X7 [Cimex lectularius]	XP_014244019	1.28737E-50	72	202
Gene11059_Transcript_13913	ubiquinol-cytochrome c rieske iron-sulfur polypeptide 1	831	gi|121543951|gb|ABM55640.1|putative ubiquinol-cytochrome c reductase, Rieske iron-sulfur polypeptide 1 [Maconellicoccus hirsutus]	ABM55640	1.9229E-123	84	276

Gene7226_Transcript_8548	protein wingless	600	gi|970905347|ref|XP_015118605.1|PREDICTED: protein wingless [Diachasma alloeum]	XP_015118605	1.133E-106	86	203
Gene9154_Transcript_11178	swi snf complex subunit smarcc2 isoform x2	2169	gi|939257713|ref|XP_014248678.1|PREDICTED: SWI/SNF complex subunit SMARCC2 isoform X2 [Cimex lectularius]	XP_014248678	0	81	647
Gene9382_Transcript_11467	apoptosis inhibitor 5	1605	gi|815816842|ref|XP_012229529.1|PREDICTED: apoptosis inhibitor 5 [Linepithema humile]	XP_012229529	8.0973E-143	66	499
Gene11474_Transcript_14436	ecdysone-induced protein 74ef isoform b	1467	gi|662184902|ref|XP_008474349.1|PREDICTED: ecdysone-induced protein 74EF isoform A [Diaphorina citri]	XP_008474349	4.14442E-87	81	216
Gene9169_Transcript_11192	peptidyl-prolyl cis-trans isomerase-like 3	486	gi|357619503|gb|EHJ72048.1|putative cyclophilin-10 [Danaus plexippus]	EHJ72048	1.62716E-96	89	161
Gene12357_Transcript_15846	serine protease p54	681	gi|546674814|gb|ERL86100.1|hypothetical protein D910_03514, partial [Dendroctonus ponderosae]	ERL86100	5.9079E-108	82	228
Gene10998_Transcript_13818	coatomer subunit delta	840	gi|939669103|ref|XP_014281485.1|PREDICTED: coatomer subunit delta [Halyomorpha halys]	XP_014281485	9.8051E-116	77	277
Gene14516_Transcript_19656	tetraspanin-17 isoform x2	762	gi|939688420|ref|XP_014288110.1|PREDICTED: tetraspanin-17 isoform X2 [Halyomorpha halys]	XP_014288110	2.79916E-56	67	197

Gene11103_Transcript_13971	insulin-like growth factor 2 mrna-binding protein partial	687	gi|646724118|gb|KDR24489.1|Insulin-like growth factor 2 mRNA-binding protein 1, partial [Zootermopsis nevadensis]	KDR24489	2.29156E-89	79	224
Gene14323_Transcript_19239	homeobox protein prospero isoform x1	2076	gi|939237231|ref|XP_014259951.1|PREDICTED: homeobox protein prospero isoform X2 [Cimex lectularius]	XP_014259951	0	69	689
Gene10292_Transcript_12769	protein crumbs-like	519	gi|501295329|dbj|BAN20858.1|crumbs [Riptortus pedestris]	BAN20858	3.13687E-59	71	174
Gene1084_Transcript_1079	armadillo segment polarity protein isoform x2	2112	gi|749735954|ref|XP_011154161.1|PREDICTED: armadillo segment polarity protein isoform X2 [Harpegnathos saltator]	XP_011154161	0	92	694
Gene7271_Transcript_8602	creb-binding protein	774	gi|972203828|ref|XP_015184076.1|PREDICTED: CREB-binding protein [Polistes dominula]	XP_015184076	3.41864E-24	53	223
Gene741_Transcript_752	heat shock protein	1338	gi|699979698|gb|AIU47025.1|heat shock protein [Phenacoccus solenopsis]	AIU47025	0	100	446
Gene147_Transcript_161	heat shock protein 70	420	gi|675275127|gb|AIL52739.1|heat shock protein 70 [Phenacoccus solenopsis]	AIL52739	6.01972E-94	100	140
Gene9220_Transcript_11266	histone deacetylase 6	501	gi|646723938|gb|KDR24372.1|Histone deacetylase 6 [Zootermopsis nevadensis]	KDR24372	1.90153E-40	64	162

Gene2523_Transcript_2639	calcitonin gene-related peptide type 1 receptor-like isoform x1	1473	gi|939279519|ref|XP_014260343.1|PREDICTED: calcitonin gene-related peptide type 1 receptor-like isoform X1 [Cimex lectularius] >gi|939279521|ref|XP_014260344.1| PREDICTED: calcitonin gene-related peptide type 1 receptor-like isoform X1 [Cimex lectularius]	XP_014260343	1.4351E-177	76	410

Supplementary information 4 SI_Table 4: Probit analysis: LD values for dsSNF7 and dsIAP at different doses in *Phenacoccus solenopsis*. The dose-mortality relationship could not be established for dsVATPase and dsAmylase due to negligible mortality at 20 to 80 μg dsRNA of respective gene.

Target gene dsRNA	LD	Effective doses	Slope	Chi-square
SNF7	LD10	17.52	0.040	0.712
	LD50	49.74		
	LD90	81.95		
IAP	LD10	2.38	0.031	0.485
	LD50	43.83		
	LD90	85.27		
